# Human marginal zone B cell development from early T2 progenitors

**DOI:** 10.1084/jem.20202001

**Published:** 2021-08-20

**Authors:** Thomas J. Tull, Michael J. Pitcher, William Guesdon, Jacqueline H.Y. Siu, Cristina Lebrero-Fernández, Yuan Zhao, Nedyalko Petrov, Susanne Heck, Richard Ellis, Pawan Dhami, Ulrich D. Kadolsky, Michelle Kleeman, Yogesh Kamra, David J. Fear, Susan John, Wayel Jassem, Richard W. Groves, Jeremy D. Sanderson, Michael G. Robson, David P. D’Cruz, Mats Bemark, Jo Spencer

**Affiliations:** 1 School of Immunology and Microbial Sciences, King’s College London, London, UK; 2 Department of Surgery, Addenbrooke's Hospital, University of Cambridge, Cambridge, UK; 3 Department of Microbiology and Immunology, Institute of Biomedicine, Sahlgrenska Academy, University of Gothenburg, Gothenburg, Sweden; 4 Biomedical Research Centre, Guy’s and St. Thomas’ NHS Trust, London, UK; 5 Liver Transplant Unit, Institute of Liver Studies, King's College Hospital, Denmark Hill, London, UK; 6 St John’s Institute of Dermatology, King’s College London, Guy’s Campus, London, UK; 7 Department of Gastroenterology, Guy’s and St Thomas’ NHS Trust, Guy’s Hospital, London, UK; 8 Department of Clinical Immunology and Transfusion Medicine, Region Västra Götaland, Sahlgrenska University Hospital, Gothenburg, Sweden

## Abstract

B cells emerge from the bone marrow as transitional (TS) B cells that differentiate through T1, T2, and T3 stages to become naive B cells. We have identified a bifurcation of human B cell maturation from the T1 stage forming IgM^hi^ and IgM^lo^ developmental trajectories. IgM^hi^ T2 cells have higher expression of α4β7 integrin and lower expression of IL-4 receptor (IL4R) compared with the IgM^lo^ branch and are selectively recruited into gut-associated lymphoid tissue. IgM^hi^ T2 cells also share transcriptomic features with marginal zone B cells (MZBs). Lineage progression from T1 cells to MZBs via an IgM^hi^ trajectory is identified by pseudotime analysis of scRNA-sequencing data. Reduced frequency of IgM^hi^ gut-homing T2 cells is observed in severe SLE and is associated with reduction of MZBs and their putative IgM^hi^ precursors. The collapse of the gut-associated MZB maturational axis in severe SLE affirms its existence in health.

## Teaser

This study identifies a developmental trajectory from human IgM^hi^ gut homing transitional 2 cells to marginal zone B cells, all stages of which are affected in patients with severe systemic lupus erythematosus.

## Introduction

Transitional (TS) B cells are the immature B cells in human blood from which all mature B cells develop. Following emigration from the bone marrow, TS B cells mature through transitional stage 1 (T1), T2, and T3 phases, when autoreactive cells are depleted (Palanichamy et al., 2009; Suryani et al., 2010; Yurasov et al., 2005).

In mice, a B cell lineage split that is dependent on B cell receptor engagement and the serine/threonine kinase Taok3 is initiated at the T1 phase (Hammad et al., 2017). This directs B cells toward marginal zone B (MZB) cell fate, requiring subsequent Notch2 cleavage by a disintegrin and metalloproteinase-containing protein 10 (ADAM10).

MZB lineage progression in humans is not clearly understood or, indeed, universally accepted. A MZB precursor (MZP) population has been proposed that undergoes terminal differentiation to MZB following Notch 2 ligation and can be discriminated from naive B cells by expression of high levels of IgM (IgM^hi^), CD24, and the glycosylation-dependent epitope CD45RB^MEM55^ (referred to here as CD45RB). An additional CD45RB^hi^ IgM^hi^ population that lacks the ABCB1 cotransporter has previously been referred to as T3′, although the relationships among this subset, MZBs, and MZPs is unclear (Bemark et al., 2013; Descatoire et al., 2014; Koethe et al., 2011; Zhao et al., 2018).

In humans, MZBs develop over the first 2 yr of life and are important for immunity against encapsulated bacteria (Weller et al., 2004). They undergo a phase of clonal expansion and receptor diversification in the germinal centers (GCs) of gut-associated lymphoid tissue (GALT; Zhao et al., 2018; Weill and Reynaud, 2020). The shared expression of MAdCAM1 between the splenic marginal zone reticular cells and GALT high endothelial venules creates the potential to recruit B cells to both sites mediated by α4β7 integrin binding (Kraal et al., 1995; Vossenkämper et al., 2013). We have described the expression of β7 integrin (used here and previously as a surrogate for α4β7) by T2 B cells in humans and observed their selective recruitment into GALT, where they become activated (Vossenkämper et al., 2013). Therefore, exposure to the GALT microenvironment could be associated with multiple stages of MZB cell development from as early as the T2 stage.

The systemic autoimmune disease systemic lupus erythematosus (SLE), in particular the severe variant lupus nephritis (LN), has markedly distorted profiles of B cell subsets in blood. The TS B cell pool is expanded, as is the B cell subset lacking both CD27 and IgD (so-called double-negative [DN] B cells; Landolt-Marticorena et al., 2011; Wei et al., 2007). Disproportionate expansion of a population of DN cells lacking expression of CD21 and CXCR5 and with up-regulated CD11c (DN2 cells) is a particular feature of LN (Jenks et al., 2018). DN2 cells may be derived from activated naive B cells (aNAV), driven by TLR7 engagement, resulting in the generation of self-reactive antibody–producing plasma cells (Jenks et al., 2018; Tipton et al., 2015). Interestingly, a recent study of a cohort of newly diagnosed patients with SLE demonstrated that MZBs may be reduced in frequency (Zhu et al., 2018). Since we have previously shown that TS B cells in SLE may have significantly reduced expression of β7 integrin, we were interested to know if this may be associated with defective MZB development and the increase in aNAV and DN2 cells.

Here, we identify bifurcation in human B cell development from the T2 stage. Cells in one branch are IgM^hi^, express β7 integrin, and are gut homing. Cells in the alternative IgM^lo^ branch have high expression of IL-4R, lower expression of β7 integrin, and do not tend to enter the gut. Transcriptomically, IgM^hi^ T2 cells share features with MZBs. B cell development progresses from T1 to MZBs via an IgM^hi^ trajectory by pseudotime analysis. IgM^hi^ T2 cells are stably IgM^hi^ in culture and have a greater tendency to make IL-10 than IgM^lo^ cells. A markedly reduced frequency of IgM^hi^β7^hi^ T2 cells was seen in patients with severe SLE, and this was associated with stark reduction in cell populations associated with MZB development. Our data link reduced access of IgM^hi^ T2 cells to GALT with defects in all stages of MZB differentiation and enables the assimilation of these elements of human MZB differentiation into a model of human B cell development.

## Results

### Segregation of B cell phenotypes from T2 through naive B cell subsets

**Figure S1.**
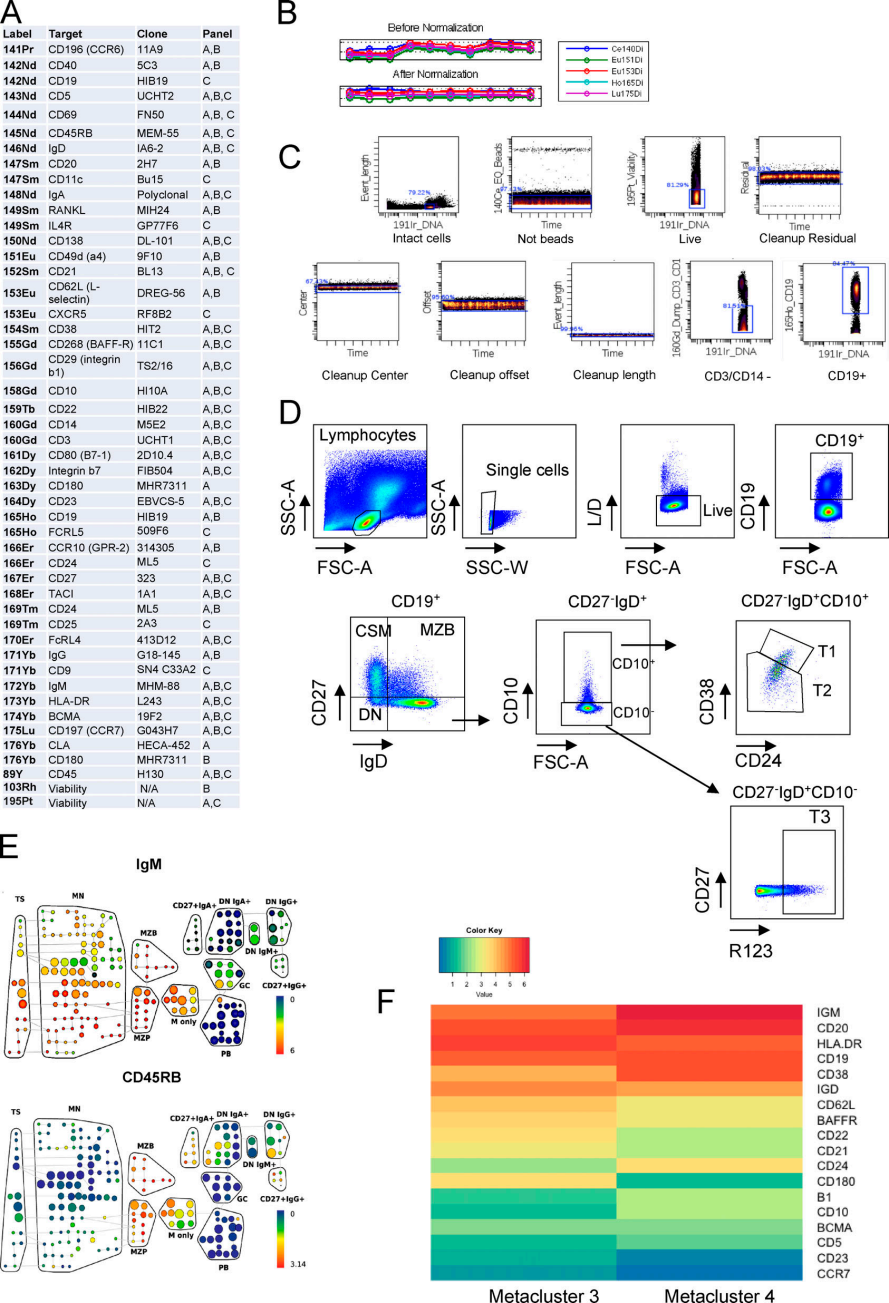
**Mass cytometry antibody panels, normalization, and gating strategy.** **(A) **Mass cytometry panel used for analysis in Fig. 1 A, Fig. 2 B, and Fig. 7 C. **(B)** Pre- and postnormalization plots of mass cytometry data used for Fig. 1, which is representative of the mass cytometry data used in Figs. 2 and 7. **(C)** Gating strategy of mass cytometry data used to identify live CD19^+^ B cells. Cleanup residual, center, offset, and length gates were not used for the data displayed in Fig. 2. **(D)** Flow cytometry plots demonstrating identification of T1 and T2 cells as CD27^−^IgD^+^CD10^+^ cells that are CD24^+++^/CD38^+++^ and CD24^+^^+^/CD38^+^^+^ respectively, T3 cells as CD27^−^IgD^+^CD10^−^R123^hi^, and naive (N) B cells as CD27^−^IgD^+^CD10^−^R123^lo^. CSM, class-switched memory. **(E)** SPADE trees demonstrating expression of IgM and CD45RB in the concatenated GALT sample (see also Fig. 2 A). **(F)** Heatmap demonstrating the median expression of panel markers from metaclusters 3 and 4 displayed in Fig. 2 B. SSC-A, side scatter area; SSC-W, side scatter width.

**Figure 1.**
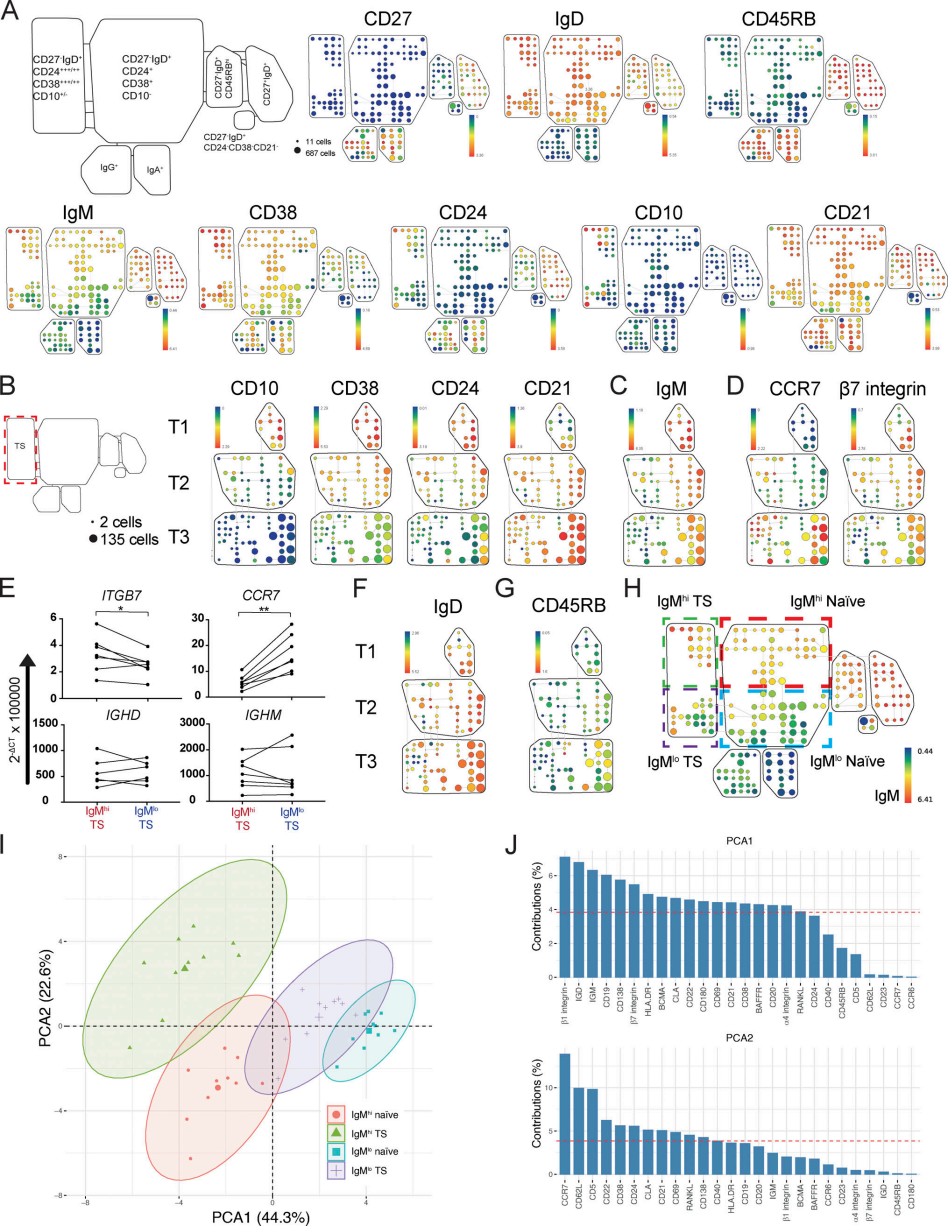
**Segregation of B cell phenotypes from T2 through naive B cell subsets.** **(A)** SPADE on viSNE plots generated using the following markers that were used to identify B cell subsets: CD10, CD24, CD27, CD38, CD45RB, IgD, IgM, IgA, and IgG. The plots are from a female HCD and are representative of 10 HCDs. Nodes represent a cluster of phenotypically similar cells, the size of a node is proportional to the number of cells represented by it, and the color indicates the median expression of a given marker. Nodes representing B cell subsets were grouped into bubbles as indicated by the schema to the left. **(B)** SPADE on viSNE plots of TS B cells exported from the TS SPADE bubble in A and generated by rerunning viSNE using all expressed B cell markers. The SPADE plots depicted are from a female HCD and are representative of 10 HCDs. TS B cell populations were defined as T1 (CD10^+^CD24^+++^CD38^+++^CD21^lo^), T2 (CD10^+^CD24^+^^+^CD38^+^^+^CD21^hi^), and T3 (CD10^−^CD24^+^CD38^+^CD21^hi^). **(C)** SPADE trees demonstrating that T2 and T3 TS B cells have prominent IgM^hi^ and IgM^lo^ subpopulations. **(D)** SPADE trees demonstrating that IgM^hi^ T2 B cells have higher expression of β7 integrin and lower expression of CCR7 than IgM^lo^ T2 B cells. **(E) **qPCR quantification of *CCR7*, *ITGB7* (β7 integrin), *IGHM*, and *IGHD* transcripts from sorted subsets expressed as 2^−ΔCT^ values relative to 18S endogenous control (paired *t* test). *, P < 0.05; **, P < 0.01. **(F)** SPADE demonstrating that IgM^hi^ T2 B cells also have high surface IgD expression. **(G)** SPADE aligns IgM^hi^ T2 cells with IgM^hi^ T3 cells with relatively high expression of CD45RB. **(H)** A SPADE on viSNE plot from A demonstrating the identification of IgM^hi^ TS and naive B cell populations. **(I)** A PCA plot generated using all expressed markers on IgM^hi^ and IgM^lo^ subsets identified in H. Data points represent individual HCD (*n* = 10) and are surrounded by 95% confidence ellipses with a larger central mean data marker. **(J)** Variable contribution bar graphs demonstrate that homing receptors are major contributors to PCA1 and PCA2 in I. The dashed red reference line represents the value where the contribution was uniform.

In mice, B cells commit to MZB differentiation soon after bone marrow emigration at the T1 stage. To seek evidence of this in humans, a deep phenotypic analysis of peripheral blood mononuclear cells (PBMCs) from healthy control donors (HCDs) was undertaken by mass cytometry (Fig. S1, A–C). Spanning tree progression of density normalized events (SPADE) on viSNE identified B cell subsets including TS B cells represented by CD27^−^IgD^+^CD24^+++/++^CD38^+++/++^ nodes that included CD10^+^ T1 and T2 cells as well as CD10^−^ T3 cells (Fig. 1 A; Qiu et al., 2011; Zhao et al., 2018). T3 cells can only be definitively distinguished from naive cells by their failure to extrude dyes such as rhodamine 123 (R123) due to lack of the ABCB1 cotransporter (Wirths and Lanzavecchia, 2005; Fig. S1 D). Since mass cytometry cannot be used to detect dye extrusion, the boundary between T3 and naive B cells was estimated to generate the TS SPADE bubble.

To perform a deep phenotypic analysis of TS B cells, events within the TS B cell bubble identified in Fig. 1, A and B, were exported and reclustered by SPADE on viSNE using all expressed panel markers and then grouped according to gradients of loss of CD10, CD38, and CD24 and gain of CD21 corresponding to T1, T2, and T3 stages of differentiation (Bemark, 2015; Fig. 1 B). The SPADE trees branched, forming two chains of nodes that each extended through the T2 and T3 SPADE bubbles with no lateral connections between the branches. Branches differed most notably in their expression of IgM (Fig. 1 C). IgM^hi^ T2 B cells also had lower expression of CCR7 but higher expression of β7 integrin than IgM^lo^ T2 cells by mass cytometry (Fig. 1 D) and quantitative PCR (qPCR; Fig. 1 E). The amount of *IGHD* or *IGHM* transcripts did not differ between IgM^hi^ and IgM^lo^ T2 cells, suggesting that posttranslational mechanisms account for variable surface immunoglobulin expression as previously reported (Bell and Goodnow, 1994; Enders et al., 2014; Fig. 1 E). Furthermore, increased IgM surface expression was not associated with a reduction in surface IgD expression as IgM^hi^ T2 B cells had high expression of both immunoglobulin isotypes (Fig. 1 F). In addition, IgM^hi^ T3 cells had higher median expression of CD24 and CD45RB than IgM^lo^ T3 cells (Fig. 1, B and G).

In the SPADE analysis of all CD19^+^ B cells, nodes representing IgM^hi^ TS B cells were continuous with IgM^hi^ naive B cells, and IgM^lo^ TS B cells were continuous with IgM^lo^ naive cells (Fig. 1, A and H). Principal-component analysis (PCA) using all markers expressed by cells in nodes identified in Fig. 1 H grouped IgM^hi^ TS B cells closest to IgM^hi^ naive B cells and most distant to IgM^lo^ TS B cells (Fig. 1 I). The major contributors to PCA1 and PCA2 in addition to IgM and IgD were mediators of cell traffic (Fig. 1 J).

Human B cells therefore segregate phenotypically as T1 cells enter into the T2 stage, forming two branches that differ in their expression of IgM and in markers of migratory potential. IgM^hi^ T2 cells resemble IgM^hi^ naive cells more closely than they resemble IgM^lo^ T2 cells with which they share markers of differentiation.

### GALT is enriched in IgM^hi^ T2 cells

**Figure 2.**
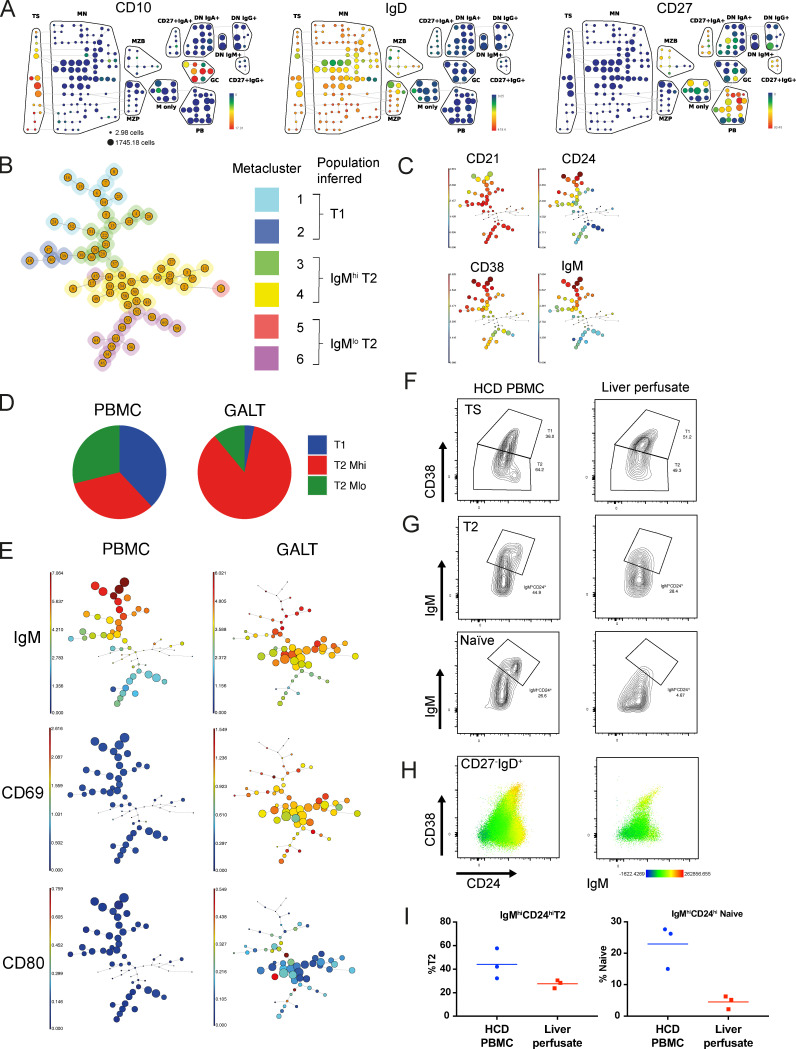
**GALT is enriched in IgM^hi^ T2 cells.** **(A)** SPADE on viSNE plots depicting the expression of B cell lineage markers used to identify T1 and T2 cells as CD27^−^IgD^+^CD10^+^ in a concatenated (*n* = 7) GALT sample. GC, IgD^−^CD10^+^; M only, IgM-only memory (CD27^+^IgD^−^IgM^+^); PB, plasmablast (IgD^−^CD38^hi^; see also Fig. S1 E). **(B)** A minimal spanning tree generated by FlowSOM run on exported events (*n* = 4,520 from each tissue) from the TS bubble in A using CD10, CD24, CD38, and IgM as clustering parameters. The tree displayed shows aggregated events from both concatenated GALT and PBMC samples. Automatic metaclustering of the FlowSOM nodes identified six metaclusters; the identity of each can be inferred by the relative expression of CD21, CD24, CD38, and IgM (see also C). **(C)** Minimal spanning trees showing expression of CD21, CD24, CD38, and IgM on a concatenated (*n* = 7) PBMC sample. Clusters represent phenotypically similar cells, their size is proportional to the number of cells contained within them, and their color indicates the median expression of a given marker. **(D)** Pie charts demonstrating the proportion of TS B cell subsets inferred from metaclusters in B confirm that GALT is enriched in IgM^hi^ T2 cells. **(E)** Minimal spanning trees demonstrating higher expression of CD69 and CD80 on GALT TS B cells. **(F)** Flow cytometry contour plots of concatenated (*n* = 3) liver perfusate samples and concatenated HCD PBMC (*n* = 3). TS B cells were gated as CD27^−^IgD^+^CD10^+^ as illustrated in Fig. S1 D. A reduced proportion of CD24^+^^+^CD38^+^^+^ T2 cells was observed in liver perfusates. **(G)** Flow cytometry plots of concatenated liver perfusate and PBMC samples demonstrating T2 cells as gated in F and naive (CD27^−^IgD^+^CD10^−^) B cells as gated in Fig. S1 D. A reduced frequency of IgM^hi^CD24^hi^ T2 and naive cells was observed in liver perfusate samples compared with HCD PBMCs. **(H)** Flow cytometry dot plots with IgM mean fluorescence intensity (MFI) overlay of concatenated PBMC (*n* = 3) and liver perfusate (*n* = 3) samples demonstrating reduced frequency of IgM^hi^CD24^hi^ TS and naive B cells in liver perfusate samples. **(I)** Scatter plots of flow cytometry data from individual samples gated as in G demonstrating reduced frequency of IgM^hi^CD24^hi^ TS and naive B cells (CD27^−^IgD^+^CD10^−^) in liver perfusate samples compared with PBMCs (median values).

Human TS B cells can home to GALT, where they become activated (Vossenkämper et al., 2013). To determine whether the high expression of β7 integrin on IgM^hi^ TS B cells is associated with selective recruitment into GALT, mass cytometry was used to compare B cells isolated from paired blood and gut biopsy specimens from individuals undergoing surveillance colonoscopies (*n* = 7; Fig. S1, A–C). TS B cells are a small subset, and due to low mononuclear cell yields from GALT biopsies, data from individual samples were concatenated. SPADE on viSNE was then used to identify CD10^+^ T1 and T2 B cells within the total CD19^+^ population (Figs. 2 A and S1 E). The undirected clustering algorithm FlowSOM (Van Gassen et al., 2015) was then used to group T1 and T2 B cells. This identified six metaclusters and the identity of each cluster was deduced from the relative expression of CD21, CD24, CD38, and IgM (Fig. 2, B and C). This demonstrated that IgM^hi^ T2 cells are enriched in GALT, whereas both T1 and IgM^lo^ T2 cells are depleted compared with PBMCs (Fig. 2, D and E). GALT T2 cells were predominantly represented by metacluster 4, while the majority of T2 cells in PBMCs were represented by metacluster 3 (Fig. 2, B and E). Metacluster 4 had higher expression of IgM, CD5, CD24, and CD38 but lower expression of CD21, CD22, CD23, and CCR7 than metacluster 3 (Fig. S1 F). Within GALT, IgM^hi^ T2 cells had higher expression of the activation markers CD69 and CD80 compared with PBMCs (Fig. 2 E).

Having observed that IgM^hi^ T2 cells are enriched in GALT, we sought confirmation of selective recruitment by asking whether this population is depleted from blood that has passed through the gut. Blood from the gut passes to the liver via the hepatic portal vein that also receives a contribution from the splenic vein. We therefore isolated lymphocytes from liver perfusion samples that would be depleted of cells that entered the gut. Using the gating strategy displayed in Fig. S1 D, liver perfusate samples were observed to be enriched in T1 cells as reported previously (Vossenkämper et al., 2013; Fig. 2 F), and CD24^hi^IgM^hi^ T2 and IgM^hi^ naive cells were depleted compared with PBMCs from HCDs (Fig. 2, G–I), consistent with their selective recruitment from blood into GALT.

### Transcriptomic analysis of IgM^hi^ and IgM^lo^ TS B cells demonstrates different upstream regulators of phenotype

**Figure S2.**
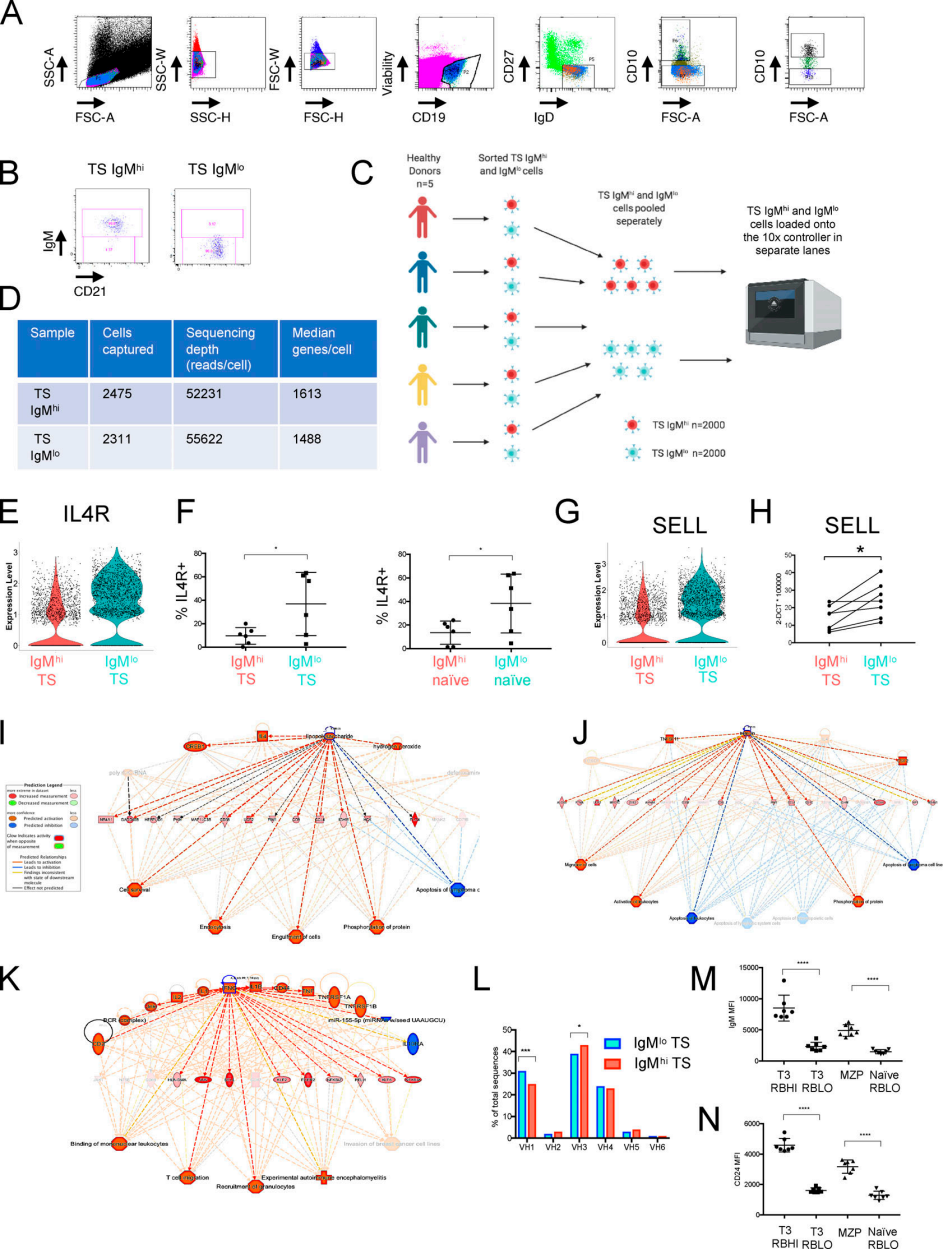
**Sort strategy, 10x Genomics workflow and validation.** **(A)** FACS sort strategy to identify IgM^hi^ and IgM^lo^ TS B cell subsets. **(B)** Purity plots of sorted IgM^hi^ and IgM^lo^ TS B cell subsets. **(C)** 10x Genomics experimental workflow detailing pooling of HCD samples. **(D)** Summary table of cell numbers captured by the 10x controller and sequencing depth of IgM^hi^ and IgM^lo^ TS B cell subsets. **(E)** Violin plot demonstrating expression of the *IL4R* gene in IgM^lo^ TS B cells. **(F)** Scatter plots of flow cytometry data demonstrating higher frequency of IL4R on IgM^lo^ compared with IgM^hi^ TS (CD27^−^IgD^+^CD10^+^) and naive (CD27^−^IgD^+^CD10^−^) cells (mean ± SD, paired *t* test). *, P < 0.05. **(G)** Violin plot demonstrating expression of the *SELL *(*CD62L*) gene in IgM^lo^ TS B cells. **(H)** qPCR confirms higher levels of the *SELL* gene transcript in IgM^hi^ TS B cells expressed as ΔCT values relative to an 18S endogenous control (paired *t* test). *, P < 0.05. **(I)** Ingenuity pathway analysis (IPA) upstream regulator plot demonstrating enrichment of LPS induced genes in IgM^hi^ TS B cells. **(J)** IPA upstream regulator plot demonstrating enrichment of retinoic acid induced genes in IgM^hi^ TS B cells. **(K)** IPA upstream regulator plot demonstrating enrichment of IFN-𝛾 induced genes in IgM^lo^ TS B cells. **(L)** Bar graphs demonstrating a lower frequency of V_H_1 and higher frequency of V_H_3 immunoglobulin variable heavy chain gene usage in IgM^hi^ TS B cells than TS IgM^lo^ cells (Chi squared test with Bonferroni correction). *, P < 0.05; ***, P < 0.001. **(M)** Scatter plot of flow cytometry data from HCD demonstrating that T3 and naive CD45RB^hi^ subsets as gated in Fig. 3 J share high IgM expression (mean fluorescence intensity [MFI] mean ± SD, paired *t* test). ****, P < 0.0001. **(N)** T3 and naive CD45RB^hi^ subsets share similar high surface expression of CD24 (MFI mean ± SD, paired *t* test). ****, P < 0.0001. FSC-H, forward scatter height; SSC-H, side scatter height.

**Figure 3.**
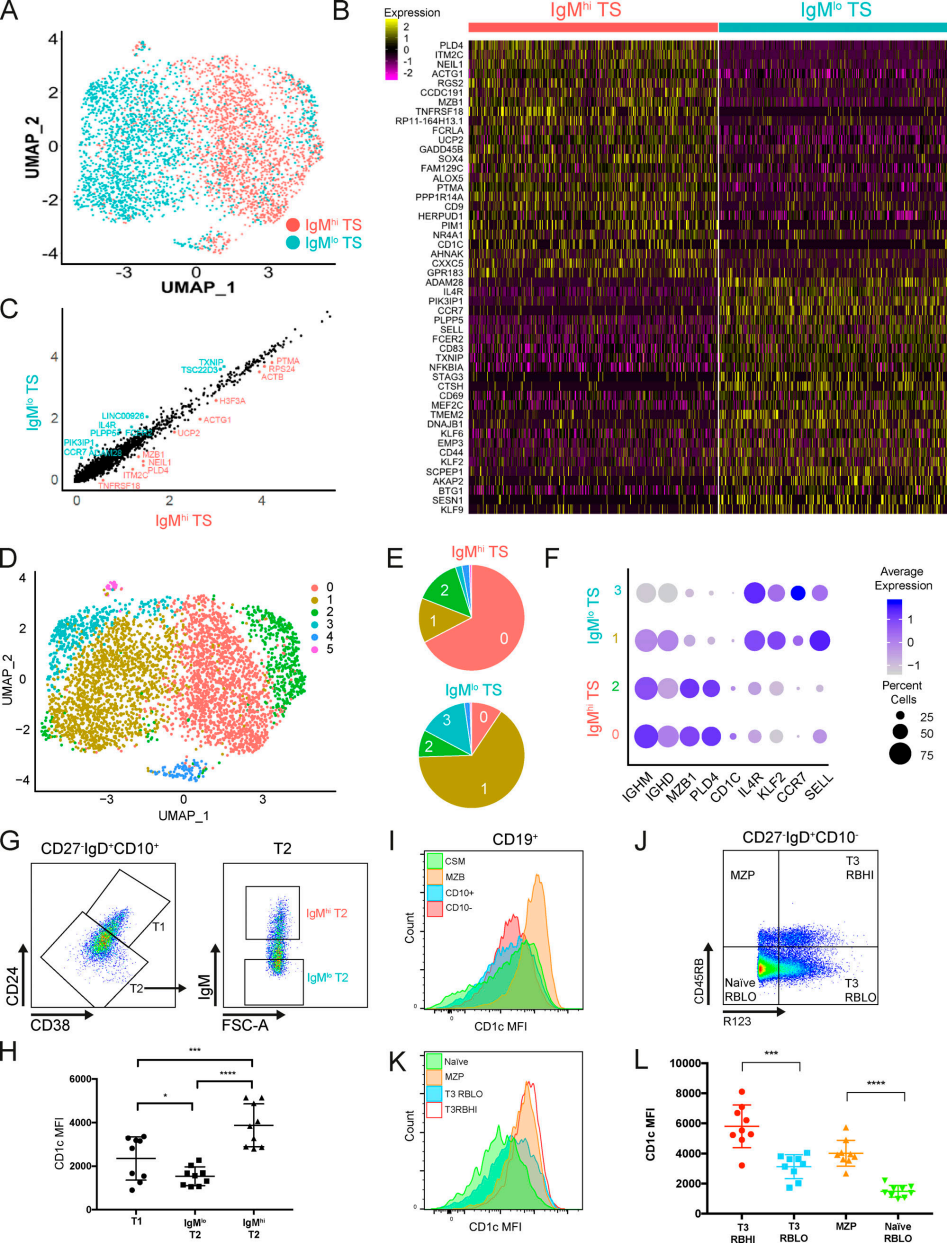
**Transcriptomic analysis of IgM^hi^ and IgM^lo^ TS B cells demonstrates different upstream regulators of phenotype.** **(A)** UMAP plot of IgM^hi^ and IgM^lo^ TS B cells from five HCDs (see Fig. S2, A–D) clustered according to differentially expressed genes identified using the Seurat SCTransform workflow (Hafmeister and Satija, 2019 *Preprint*). **(B)** Heatmap of selected genes from the top 60 differentially expressed genes in IgM^hi^ and IgM^lo^ TS B cells. **(C)** Scatter plot demonstrating genes differentially expressed in IgM^hi^ and IgM^lo^ TS B cells. **(D)** A PCA-based approach based on differentially expressed genes identified six clusters among the IgM^hi^ and IgM^lo^ TS B cells that were demonstrated by UMAP. **(E)** Quantification of the frequency of IgM^hi^ or IgM^lo^ TS B cells within the clusters demonstrated by UMAP in D reveals that IgM^hi^ TS B cells dominate in clusters 0 and 2 and IgM^lo^ TS B cells in clusters 1 and 3. **(F)** Dot plot demonstrating expression of selected genes within clusters 0–3. **(G)** Flow cytometry dot plots demonstrating CD27^−^IgD^+^CD10^+^ cells as gated in Fig. S1 D. T2 cells were gated as CD24^++^CD38^++^ and IgM^hi^ and IgM^lo^ subsets as 30% of cells with the highest and lowest expression of IgM respectively. **(H)** Scatter plots demonstrating CD1c mean fluorescence intensity (MFI) in T1, IgM^hi^, and IgM^lo^ T2 B cells gated in G (mean ± SD, paired *t* test). *, P < 0.05; ***; P < 0.001; ****, P < 0.0001. **(I)** Histograms demonstrating CD1c MFI in B cell subsets as gated in Fig. S1 D. **(J)** Dot plot of flow cytometry data demonstrating CD27^−^IgD^+^CD10^−^ cells as gated in Fig. S1 D. Differential expression of CD45RB and R123 allowed the identification of CD45RB^hi^ T3 (R123^+^) and MZP (R123^−^) cells. **(K)** A histogram showing CD1c MFI on subsets as gated in J. **(L)** Dot plots demonstrating CD1c MFI on subsets gated in J (mean ± SD, paired *t* test). In J–L, RBHI refers to CD45RB^hi^ and RBLO refers to CD45RB^lo^. ***, P < 0.001; ****, P < 0.0001. FSC-A, forward scatter area.

Having demonstrated contrasting surface phenotypes and migratory capacity of IgM^hi^ and IgM^lo^ TS B cells, we next sought to identify transcriptomic features differing between them and to gain insight into inducers and regulators of these subsets by single-cell RNA sequencing. IgM^hi^ and IgM^lo^ TS B cells from five HCDs were sorted by FACS and pooled, and gene expression libraries were prepared using a 10x Genomics single-cell 5′ gene expression workflow (Fig. S2, A–D). In total, 14,499 genes were identified in 4,268 cells after quality filtering. The nonlinear dimension reduction algorithm UMAP (uniform manifold approximation and projection; Becht et al., 2018) was run on differentially expressed genes and demonstrated discreet clustering of IgM^hi^ and IgM^lo^ TS B cells (Fig. 3 A). Selected genes from the top 60 differentially expressed genes are illustrated in Fig. 3, B and C. Transcripts encoding *CD1C* and *MZB1* that are expressed by MZB cells were among the most abundantly expressed genes in IgM^hi^ TS B cells. The lupus risk allele and regulator of TLR9 responses *PLD4* was the most highly differentially expressed gene in IgM^hi^ TS B cells (Gavin et al., 2018). Undirected clustering of pooled IgM^hi^ or IgM^lo^ TS B cells generated clusters that contained predominantly IgM^hi^ or IgM^lo^ TS B cells (Fig. 3, D and E) that shared enrichment of the genes expressed by these cell subsets (Fig. 3, B and F). High expression of IL4R by IgM^lo^ TS and IgM^lo^ naive B cells was confirmed by flow cytometry (Fig. S2, E and F). Importantly, *KLF2,* which drives murine follicular B cell development, was up-regulated in IgM^lo^ TS B cells (Hart et al., 2011). *CCR7* was up-regulated in IgM^lo^ TS B cells supporting the higher surface expression that was evident in the mass cytometry analysis. Higher abundance of transcripts encoding L-selectin by IgM^lo^ TS B cells was also confirmed by qPCR using sorted populations (Fig. S2, G and H).

Ingenuity pathway analysis demonstrated enrichment of retinoic acid receptor and LPS-induced genes in IgM^hi^ TS B cells (Fig. S2, I and J). IgM^lo^ TS B cells were enriched in genes induced by IFN-γ, IL-1, and IL-2 (Fig. S2 K). IgM^hi^ TS B cells used less V_H_1 and more V_H_3 than IgM^lo^ TS B cells, consistent with published profiles of the MZB repertoire (Bagnara et al., 2015; Fig. S2 L).

IgM^hi^ and IgM^lo^ TS B cells therefore have distinct transcriptomes. IgM^lo^ cells are selectively enriched in genes encoding peripheral circulation and inhibition of MZB cell fate, whereas IgM^hi^ cells have gene expression signatures and IGHV gene family usage linking them to MZB cells.

The abundance of *CD1C* transcripts in IgM^hi^ TS B cells was of particular interest, because CD1c is characteristically highly expressed by human MZBs (Weller et al., 2004). Consistent with the transcriptomic profile, CD1c surface expression was higher on IgM^hi^ than IgM^lo^ TS B cells (Fig. 3, G and H). As previously reported, CD1c expression was high on MZBs (Fig. 3 I) as well as on MZP and CD45RB^hi^ T3 cells (previously referred to as T3′) that have been linked to MZB development (Bemark et al., 2013; Descatoire et al., 2014; Koethe et al., 2011; Zhao et al., 2018). MZP and CD45RB^hi^ T3 cells were defined by the phenotype CD27^−^IgD^+^CD10^−^CD45RB^hi^ with expression of the ABCB1 cotransporter or not, respectively (Fig. S1 D and Fig. 3, J–L). Cells that express the ABCB1 cotransporter extrude R123 and are therefore identified as R123^lo^ cells in this analysis. Both subsets share high expression of IgM and CD24 (Fig. S2, M and N).

### Lineage progression from IgM^hi^ TS B cells through to MZB cells

The shared surface properties of IgM^hi^ TS with IgM^hi^ naive B cells (Fig. 1 I), the enrichment of transcripts considered characteristic of MZBs in IgM^hi^ TS B cells (Fig. 3, B and F), and shared high expression of CD1c by IgM^hi^ TS with MZBs and other B cell subsets associated with MZB development (Fig. 3, H and L) all support the existence of an IgM^hi^ MZB differentiation pathway that begins during TS B cell development. We investigated this further by performing pseudotime trajectory analysis of single-cell RNA-sequencing data from HCD B cells from blood.

**Figure S3.**
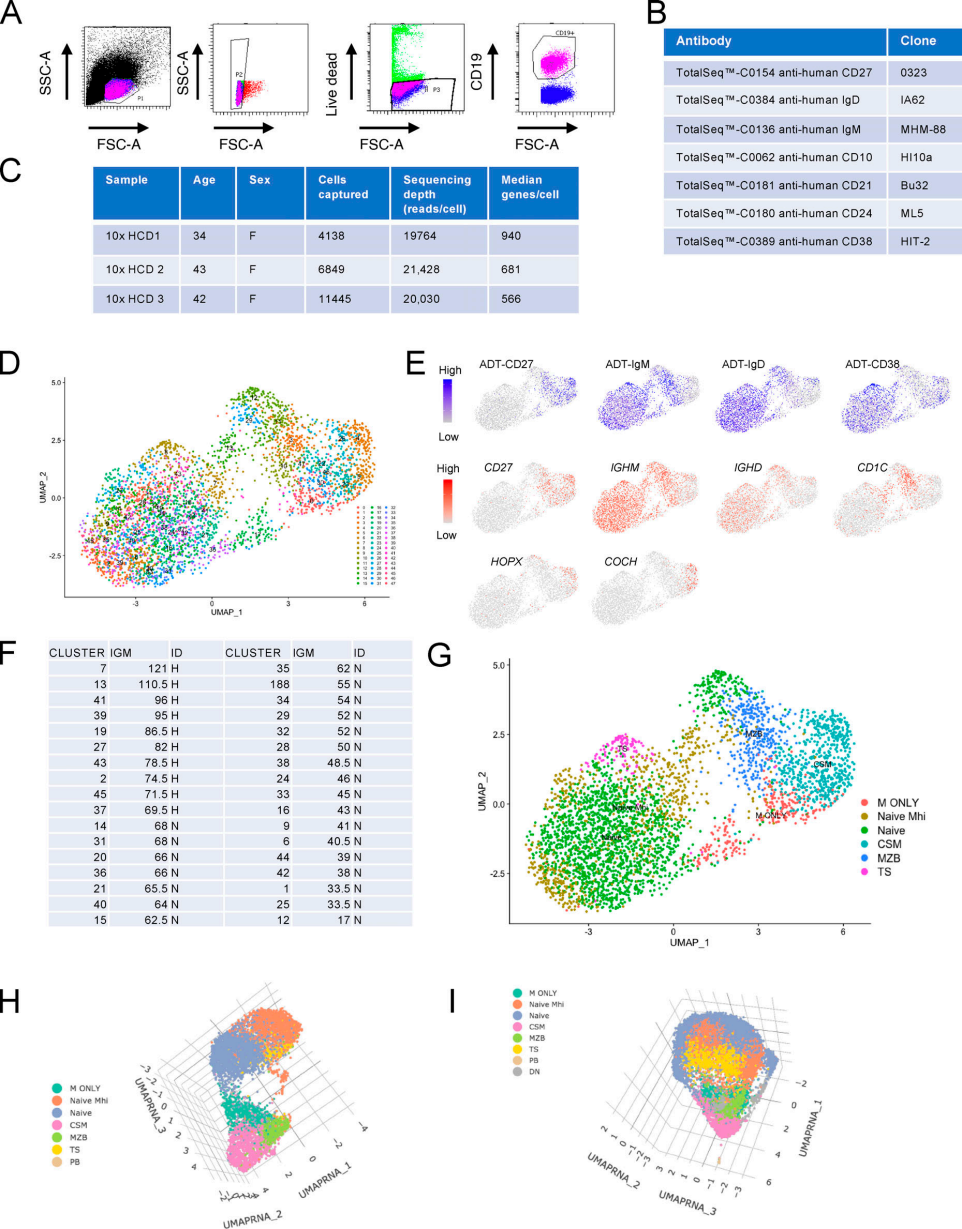
**Sort strategy, 10x Genomics workflow and identification of B cell subsets represented by UMAP clusters in 10x HCD1.** **(A)** Gating strategy to sort live CD19^+^ cells. **(B)** TotalSeq antibodies and clones used for surface labeling of CD19^+^ B cells. **(C)** Demographic details of HCDs, cells captured, and sequencing depth. **(D)** UMAP plot demonstrating clusters generated from a PCA run on 2,000 differentially expressed genes from 10x HCD1. **(E)** Feature plots demonstrating lineage defining ADT (CITE-seq antibody) and transcript signal overlay on the UMAP plot. **(F)** Table of median IgM (IGM) expression within clusters representing naive cells (CD27^−^IgD^+^CD38^int^). The top 30% of clusters were designated as IgM^hi^ and designated H in the column labeled ID. The remainder of clusters that were not IgM^hi ^are designated N. **(G)** Merged and pseudocolored clusters representing B cell subsets defined by ADT and gene signal of lineage defining targets. CSM, class-switched memory. **(H)** A 3D UMAP plot demonstrating merged and pseudocolored clusters representing B cell subsets from 10x HCD2. **(I)** A 3D UMAP plot demonstrating merged and pseudocolored clusters representing B cell subsets from 10x HCD3. FSC-A, forward scatter area; SSC-A, side scatter area.

CD19^+^ B cells were sorted from PBMCs of three HCD (Fig. S3 A) and surface labeled with Total-Seq-C antibodies before capture on the 10x Genomics chromium controller (Fig. S3 B). Gene expression and antibody detection tag (ADT) libraries were then prepared according to the manufacturer’s instructions and sequenced on an Illumina HiSeq High Output platform (Fig. S3 C).

Data from single HCDs were initially analyzed individually. UMAP plots were used to visualize clusters and identify the B cell subsets they corresponded to by overlaying signal from lineage-defining transcripts and CITE-seq (cellular indexing of transcriptomes and epitopes by sequencing) antibodies (Fig. S3, D and E). TS B cells were identified as CD27^−^IgD^+^ clusters with high surface expression of CD38. Of the remaining CD27^−^IgD^+^ clusters that represented naive cells, those with the top 30% of median IgM ADT signal were designated IgM^hi^ (Fig. S3, D–G). Note that because identification of MZPs and CD45RB^hi^ T3 would require reagents that are incompatible with this method (Fig. 3 J), they will be included in the IgM^hi^ naive cell groups in this analysis. CD27^+^IgD^+^ clusters that were enriched in *CD1C* transcripts were designated as MZBs. CD27^+^IgD^−^IgM^+^ clusters were designated “IgM-only” cells, and CD27^+^IgD^−^IgM^−^ clusters enriched in *HOPX* and *COCH* transcripts were designated as class-switched memory B cells (Descatoire et al., 2014; Fig. S3, D–G).

**Figure 4.**
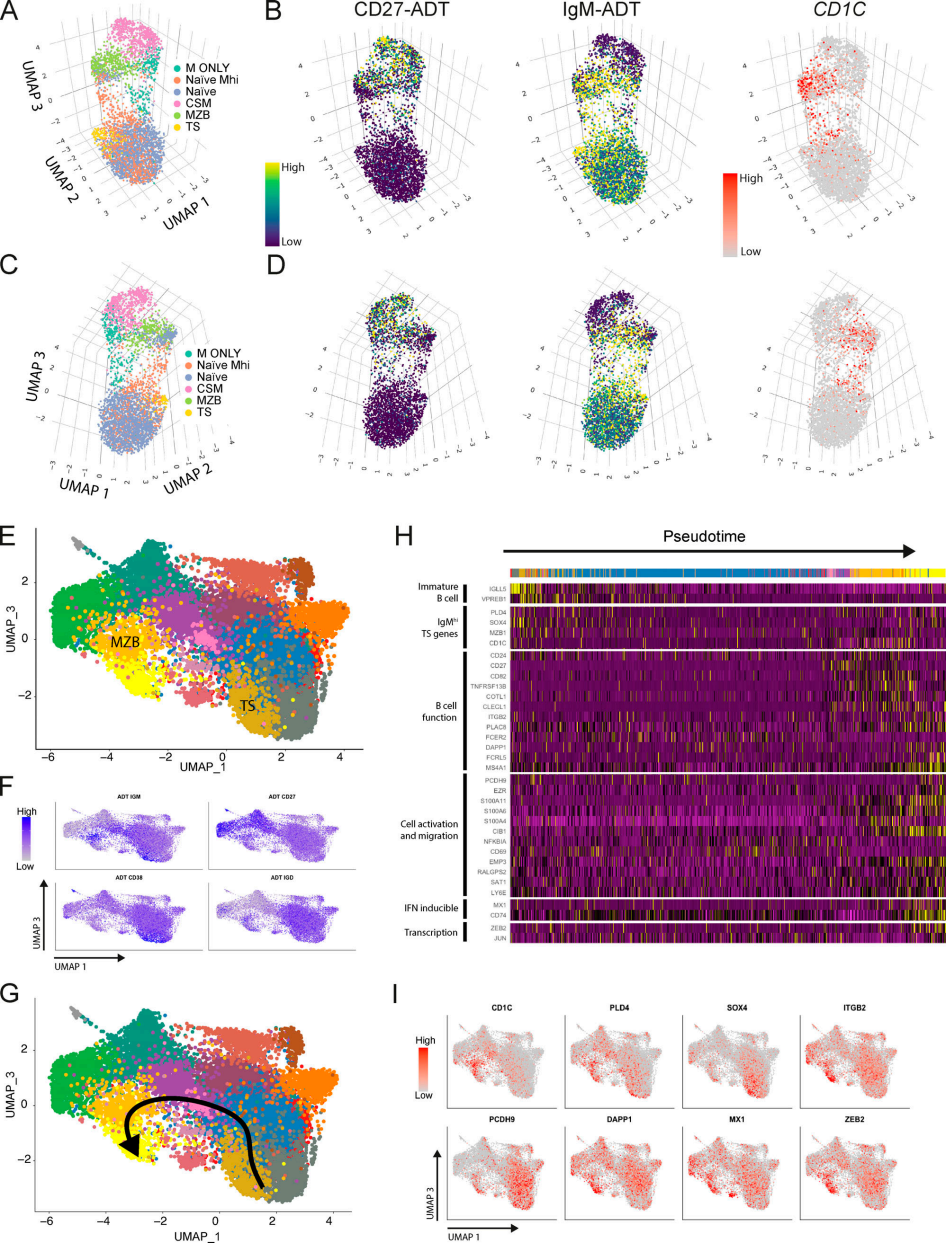
**Lineage progression from IgM^hi^ TS B cells through to MZBs.** **(A)** A 3D UMAP plot of CD19^+^ cells from a HCD (10x HCD1, see Fig. S3 C) generated from a PCA run on 2,000 differentially expressed genes. Clusters were merged and pseudocolored according to the B cell subsets they represent as described in Fig. S3 (D–G). **(B)** CD27 and IgM ADT and *CD1C* gene signal overlay on the 3D UMAP plot displayed in A. **(C)** The 3D UMAP plot displayed in A viewed using different UMAP axis coordinates. **(D)** CD27 and IgM ADT and *CD1C* gene signal overlay on the 3D UMAP projection in C. **(E)** A UMAP plot generated by integrating 3 HCD 10x datasets (see Fig. S3 C) and generated from a PCA run on 2,000 variably expressed genes. **(F)** Feature plots demonstrating overlay of ADT signal on the UMAP plot from E enables identification of clusters representing TS and MZB cells. **(G)** A Slingshot developmental trajectory overlaid onto the UMAP plot from E demonstrating developmental progression from clusters representing TS B cells to MZBs via IgM^hi^ naive B cells. **(H)** A heatmap of selected genes from the top 100 most differentially expressed genes along the Slingshot trajectory demonstrated in G. **(I)** Feature plots demonstrating overlay of gene signal from the differentially expressed genes along the Slingshot trajectory identified in G.

Three-dimensional (3D) UMAP plots were then used to better visualize the spatial relationship between these B cell subsets (Fig. 4, A–D; and Fig. S3, H and I). This demonstrated clear separation of CD27^+^ and CD27^−^ “islands” of cells (Fig. 4, A–D; Fig. S3, H and I; and Videos 1, 2, and 3). In all three HCDs, two distinct cellular “bridges” linked the CD27^−^ and CD27^+^ islands in the plot (Fig. 4, A and B; Fig. S3, H and I; and Videos 1, 2, and 3). In each HCD, an IgM^hi^ bridge that was enriched in cells with *CD1C* transcripts linked the CD27^−^ island to MZBs (Fig. 4, A–D; and Fig. S3, H and I). In contrast, IgM-only cells were connected to the CD27^−^ island by naive cells with lower expression of IgM.

**Video 1.** **Rotation of the 3D UMAP plot demonstrating B cell subsets from 10x HCD1 as depicted in ****Fig. 4 A****. **Playback speed, 10 frames per second (see also **Fig. S3, D–G**).

**Video 2.** **Rotation of the 3D UMAP plot demonstrating B cell subsets from 10x HCD2 as demonstrated in ****Fig. S3 H****. **Playback speed, 10 frames per second.

**Video 3.** **Rotation of the 3D UMAP plot demonstrating B cell subsets from 10x HCD3 as demonstrated in ****Fig. S3 I****. **Playback speed, 10 frames per second.

Having visualized the juxtaposition of IgM^hi^ naive cells with MZB in UMAP clusters we next used the Slingshot tool for pseudotime trajectory analysis. Data from the three HCD were normalized and integrated. UMAP plots were used to identify clusters representing CD27^−^CD38^hi^CD24^hi^ TS B cell and CD27^+^IgD^+^IgM^+^ MZB subsets by overlay of CD27, IgM, IgD and CD38 ADT signal (Fig. 4, E and F). The TS B cell cluster was selected as the starting point for analysis of pseudotime transitions in Slingshot. Importantly, end points were not specified.

Slingshot identified an IgM^hi^ pseudotime trajectory from TS B cells that passed through the MZB cluster via IgM^hi^ naive B cells (Street et al., 2018; Fig. 4 G and Video 4). Among the 100 most differentially expressed genes along this trajectory were *PLD4*, *CD1C*, *SOX4*, and *MZB1*, which were previously identified as differentially expressed between IgM^hi^ and IgM^lo^ TS B cells (Fig. 3, B and F; and Fig. 4, H and I). Analysis of gene expression by cells along the trajectory demonstrated progressive down-regulation of *IGLL5* and *VPREB1* markers of B cell immaturity (Fig. 4 H). Up-regulated in the terminal stages of the trajectory were genes encoding proteins implicated in cell adhesion, including *ITGB2*, *PCDH9*, and activation, including *DAPP1* (Fig. 4, H and I). The final cluster in the pseudotime trajectory was enriched in the IFN-regulated gene *MX1* and the transcription factor *ZEB2* (Fig. 4, H and I). IFN-induced genes as well as *DAPP1* and *FCRL5* are highly expressed by DN2 cells, although the relationship of this subset with MZB is not known (Jenks et al., 2018). Pseudotime analysis of HCD PBMCs therefore identified an IgM^hi^ developmental trajectory from TS B cells to MZBs.

**Video 4.** **Rotation of 3D UMAP plot as depicted in ****Fig. 4 G**** with overlay of IgM ADT signal demonstrating that the Slingshot trajectory passes through IgM^hi^ naive B cells. **Playback speed, 10 frames per second.

### IgM^hi^ and IgM^lo^ TS B cells differ functionally and in their potential to differentiate

**Figure 5.**
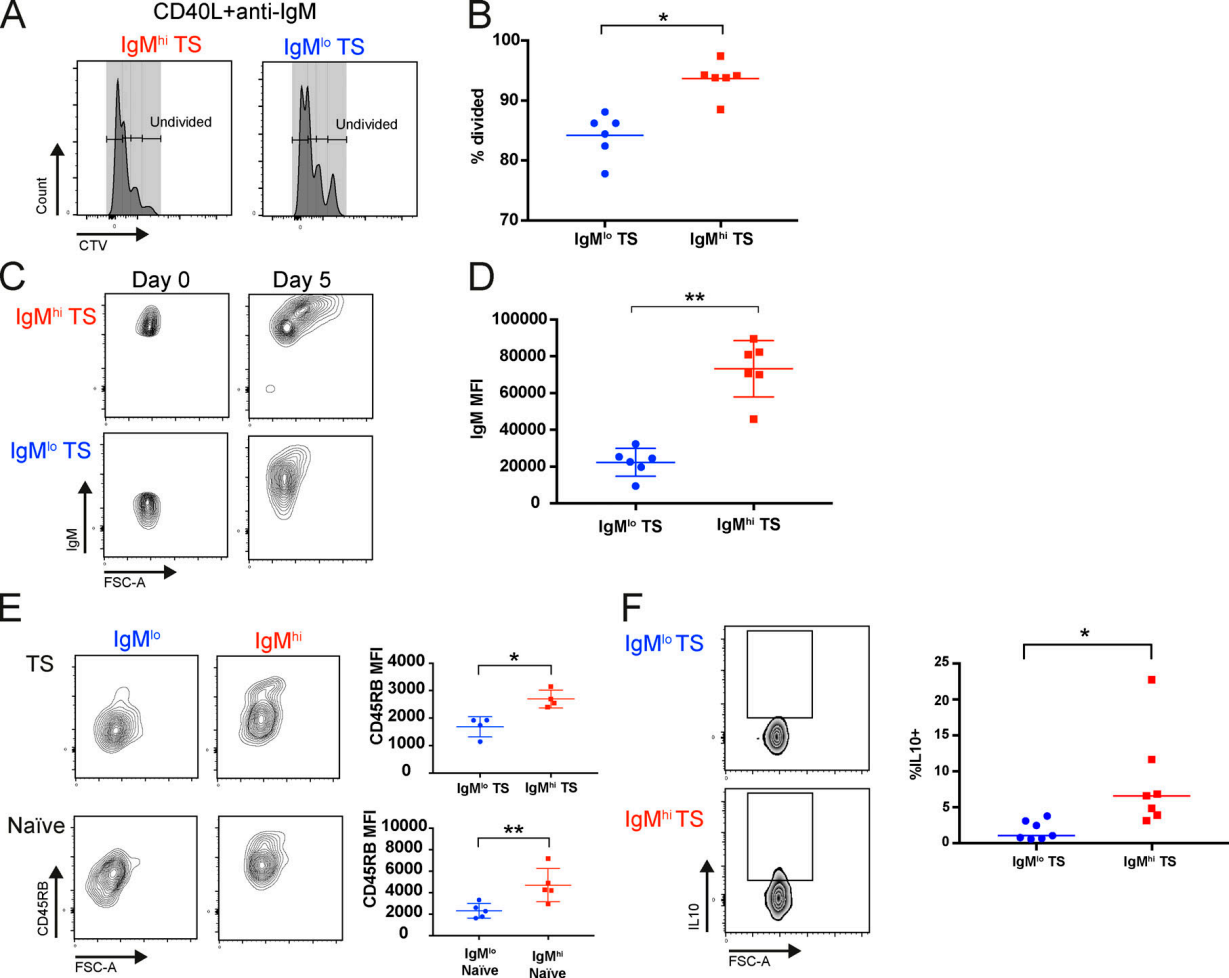
**IgM^hi^ and IgM^lo^ TS B cells differ functionally and in their potential to differentiate.** **(A)** Histograms of flow cytometry data demonstrating cell trace violet (CTV) dilution following 5 d of culture with CD40L + anti-IgM in IgM^hi^ and IgM^lo^ TS B cells from an HCD. **(B)** Scatter plots demonstrating increased proliferation of IgM^hi^ TS compared with IgM^lo^ TS B cells when stimulated with CD40L + anti-IgM for 5 d (medians, Wilcoxon test). *, P < 0.05. **(C)** Flow cytometry contour plots demonstrating that an IgM^hi^ phenotype is maintained on IgM^hi^ TS B cells after CpG stimulation for 5 d. **(D)** Scatter plots demonstrating that an IgM^hi^ phenotype is maintained on IgM^hi^ TS B cells after CpG stimulation for 5 d (mean ± SD, paired *t* test). **, P < 0.01. **(E)** Flow cytometry contour plots and scatter plots demonstrating greater up-regulation of CD45RB by IgM^hi^ than IgM^lo^ TS and naive (CD27^−^IgD^+^CD10^−^) B cell subsets at day 5 culture with CpG (mean ± SD, paired *t* test). *, P < 0.05; **, P < 0.01. **(F)** Flow cytometry contour plots and scatter plots demonstrating higher frequency of IL10 expressing cells among IgM^hi^ TS B cells following 6-h stimulation with PMA/ionomycin (median, Wilcoxon test). *, P < 0.05.

We next determined if IgM^hi^ and IgM^lo^ TS B cells that have different cell surface and transcriptomic characteristics maintain their relative levels of IgM expression in vitro following stimulation and if they differ functionally. Initially, proliferation in response to anti-IgM in the presence of CD40L was measured. IgM^hi^ TS B cells proliferated more than IgM^lo^ cells in response to anti-IgM (Fig. 5, A and B). Next, we investigated the response of IgM^hi^ and IgM^lo^ TS B cells to the TLR9 agonist CpG, which has been proposed to drive MZB differentiation (Guerrier et al., 2012). In culture, CpG increased surface expression of IgM on both IgM^hi^ and IgM^lo^ B cells. However, IgM^hi^ cells remained IgM^hi^ compared with the IgM^lo^ cells (Fig. 5, C and D). Furthermore, culture with CpG resulted in greater up-regulation of CD45RB on IgM^hi^ TS and IgM^hi^ naive B cells than IgM^lo^ TS and IgM^lo^ naive B cells, consistent with adoption of an MZP-like phenotype (Fig. 5 E).

A subpopulation of human cells with a TS B cell phenotype are regulatory, and murine IL-10–producing B regulatory (B reg) cells are T2 marginal zone progenitor cells, and the gut is important for their induction (Blair et al., 2010; Pillai et al., 2005; Rosser et al., 2014). We therefore investigated the capacity of IgM^hi^ TS B cells to produce IL-10. Following 6-h stimulation with PMA and ionomycin, IgM^hi^ TS B cells produced significantly more IL-10 than IgM^lo^ cells (Fig. 5 F), inferring greater regulatory capacity of this subset.

### MZB cell differentiation is defective in patients with severe SLE

We have previously observed reduced frequencies of circulating TS B cells expressing β7 integrin in a subset of SLE patients, implying reduced potential for TS B cells to access GALT in these cases. Data presented here implicate GALT as an important site for MZB differentiation, and MZB depletion has been reported in SLE (Rodríguez-Bayona et al., 2010; Zhu et al., 2018). Hence, we sought to determine whether the stages of our proposed MZB differentiation pathway were affected in SLE.

**Figure S4.**
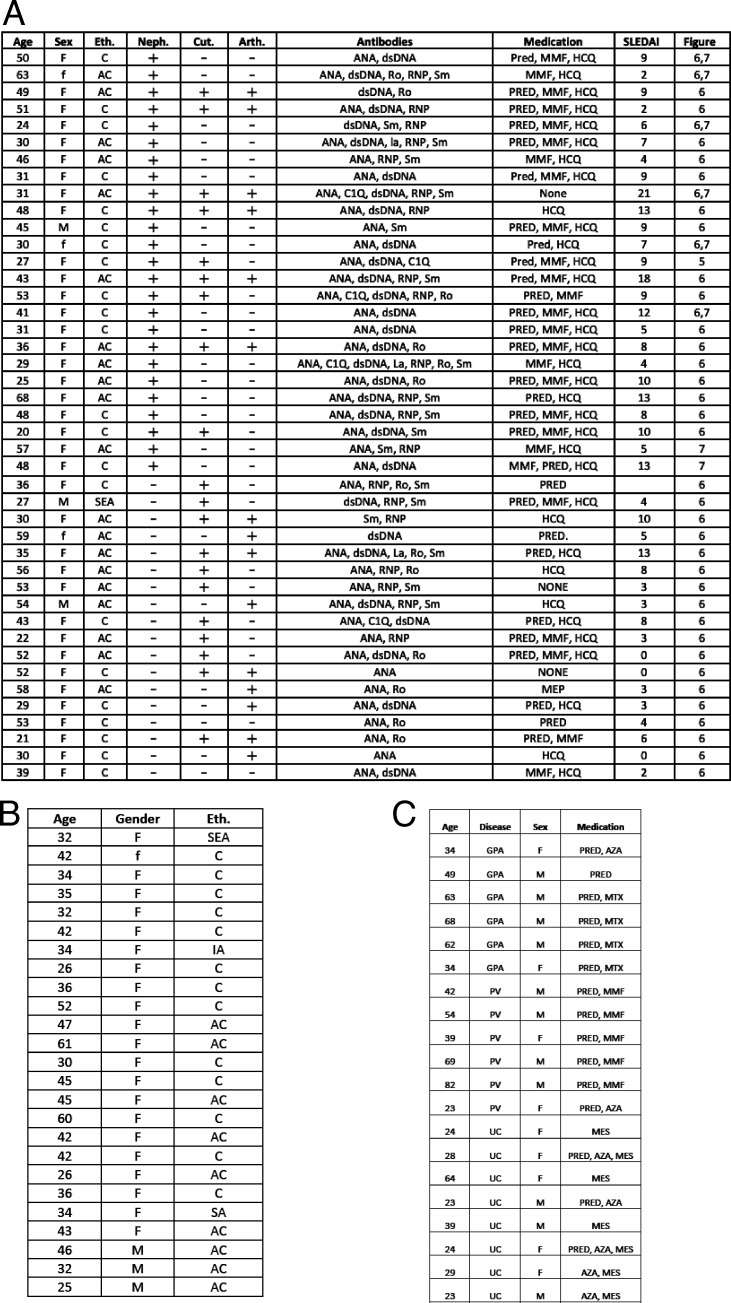
**Patient and HCD demographic tables.** **(A)** Table of SLE patient demographic data used for Figs. 6 and 7. AC, African Caribbean; Arth., inflammatory arthritis; C, Caucasian; Cut., cutaneous lupus; dsDNA, double-stranded DNA; Eth., ethnicity; HCQ, hydroxychloroquine; MMF, mycophenolate mofetil; Neph., LN; PRED, prednisolone; SEA, Southeast Asian; ANA, antinuclear antibodies; RNP, ribonucleoprotein; La, Ro, and Sm are examples of ribonuclear protein autoantigens. **(B)** Table of HCD demographic data used in Fig. 6. IA, Indian Asian. **(C)** Table of demographic data of patients with other autoimmune diseases. AZA, azathioprine; GPA, granulomatosis with polyangiitis; MES, mesalazine; MTX, methotrexate; UC, ulcerative colitis.

**Figure 6.**
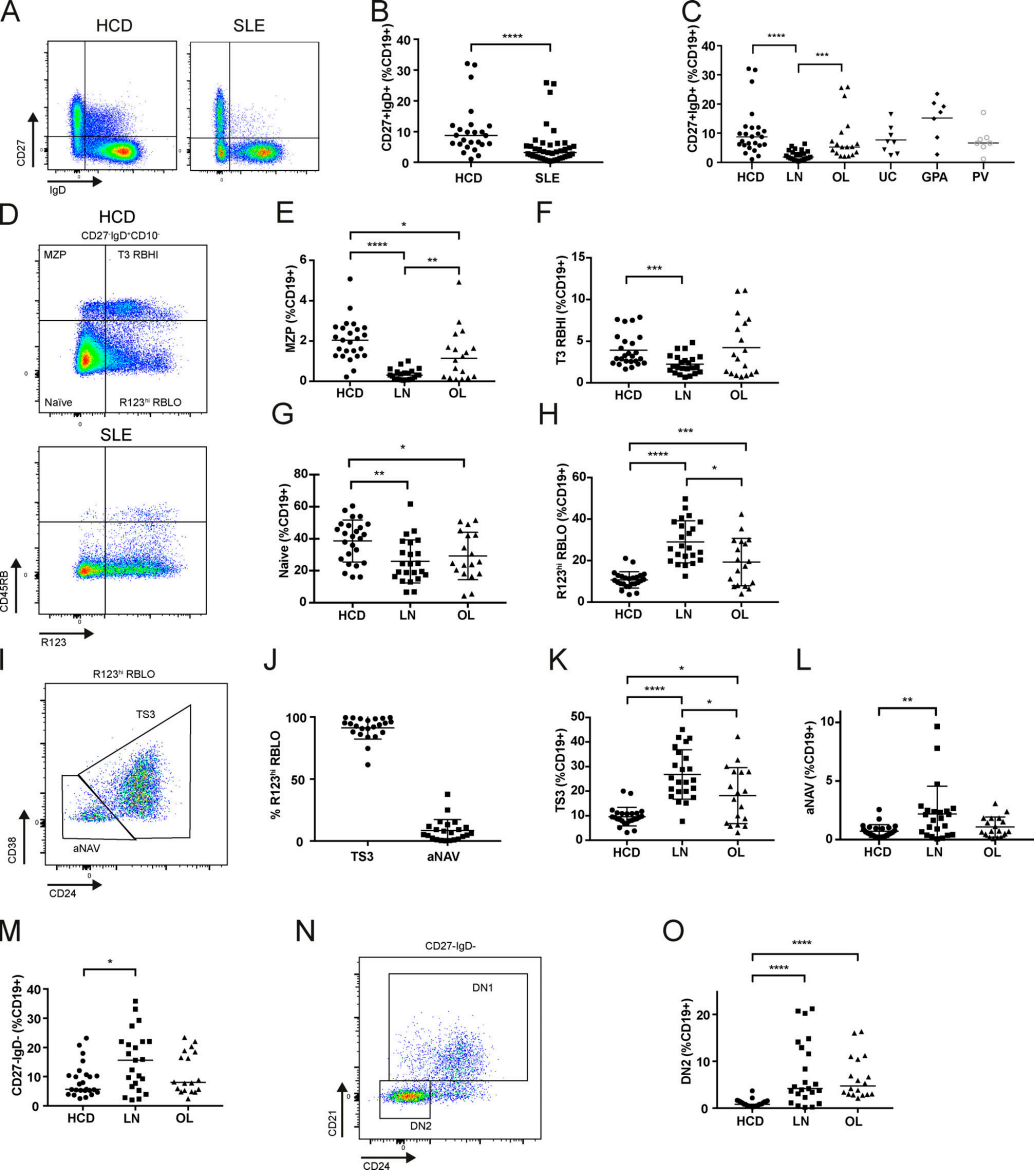
**MZB cell differentiation is defective in patients with severe SLE.** **(A)** Flow cytometry dot plots and scatter plots demonstrating reduced frequency of CD27^+^IgD^+^ (MZB) cells in a patient with SLE compared with an HCD. **(B)** Scatter plots of flow cytometry data demonstrating reduced frequency of MZB in patients with SLE compared with HCDs (medians, Mann–Whitney test). ****, P < 0.0001. **(C)** Scatter plots of flow cytometry data demonstrating reduced MZB frequency in LN, other lupus subtypes (OL) but not in ulcerative colitis (UC), granulomatosis with polyangiitis (GPA), and PV (medians, Mann–Whitney test). ***, P < 0.001; ****, P < 0.0001. **(D)** Flow cytometry dot plots of a HCD and an SLE patient demonstrating identification of T3 (R123^hi^) and naive (R123^lo^) subsets with high and low expression of CD45RB. Stark reduced frequency of CD45RB^hi^ T3 and naive (MZP) populations is evident in SLE. **(E)** Scatter plot of flow cytometry data demonstrating reduced frequency of MZP (CD45RB^hi^R123^lo^) cells in LN patients (medians, Mann–Whitney test). *, P < 0.05; **, P < 0.01; ****, P < 0.0001. **(F)** Scatter plot of flow cytometry data demonstrating reduced frequency of CD45RB^hi^ T3 (R123^hi^) cells in LN patients (medians, Mann–Whitney test). ***, P < 0.001. **(G)** Scatter plot of flow cytometry data demonstrating reduced frequency of naive (CD45RB^lo^R123^lo^) cells in LN patients (mean ± SD, unpaired *t* test). *, P < 0.05; **, P < 0.01. **(H)** Scatter plot of flow cytometry data demonstrating enrichment of CD45RB^lo^R123^hi^ cells in LN patients (mean ± SD, unpaired *t* test). *, P < 0.05; ***, P < 0.001; ****, P < 0.0001. **(I)** Gating strategy to distinguish T3 (TS3) and aNAV cell subsets among R123^hi^CD45RB^lo^ cells on the basis of CD24 and CD38 expression. **(J)** Scatter plot demonstrating that R123^hi^CD45RB^lo^ cells were mostly T3 cells as gated in I (mean ± SD). **(K)** Scatter plot demonstrating increased frequency of T3 cells in OL and LN patients (mean ± SD, unpaired *t* test). *, P < 0.05; ****, P < 0.0001. **(L)** Scatter plot demonstrating increased frequency of aNAV cells in OL and LN patients (mean ± SD, unpaired *t* test). **, P < 0.01. **(M)** Scatter plots showing the proportion of CD27^−^IgD^−^ B cells as gated in A demonstrate increased frequency of this population in LN (medians, Mann–Whitney test). *, P < 0.05. **(N)** Flow cytometry dot plot demonstrating the identification of DN1 and DN2 cells based on expression of CD21 and CD24. **(O)** Scatter plot showing that DN2 cells as gated in N were more abundant in LN and OL patients than in HCD (medians, Mann–Whitney test). ****, P < 0.0001.

**Figure S5.**
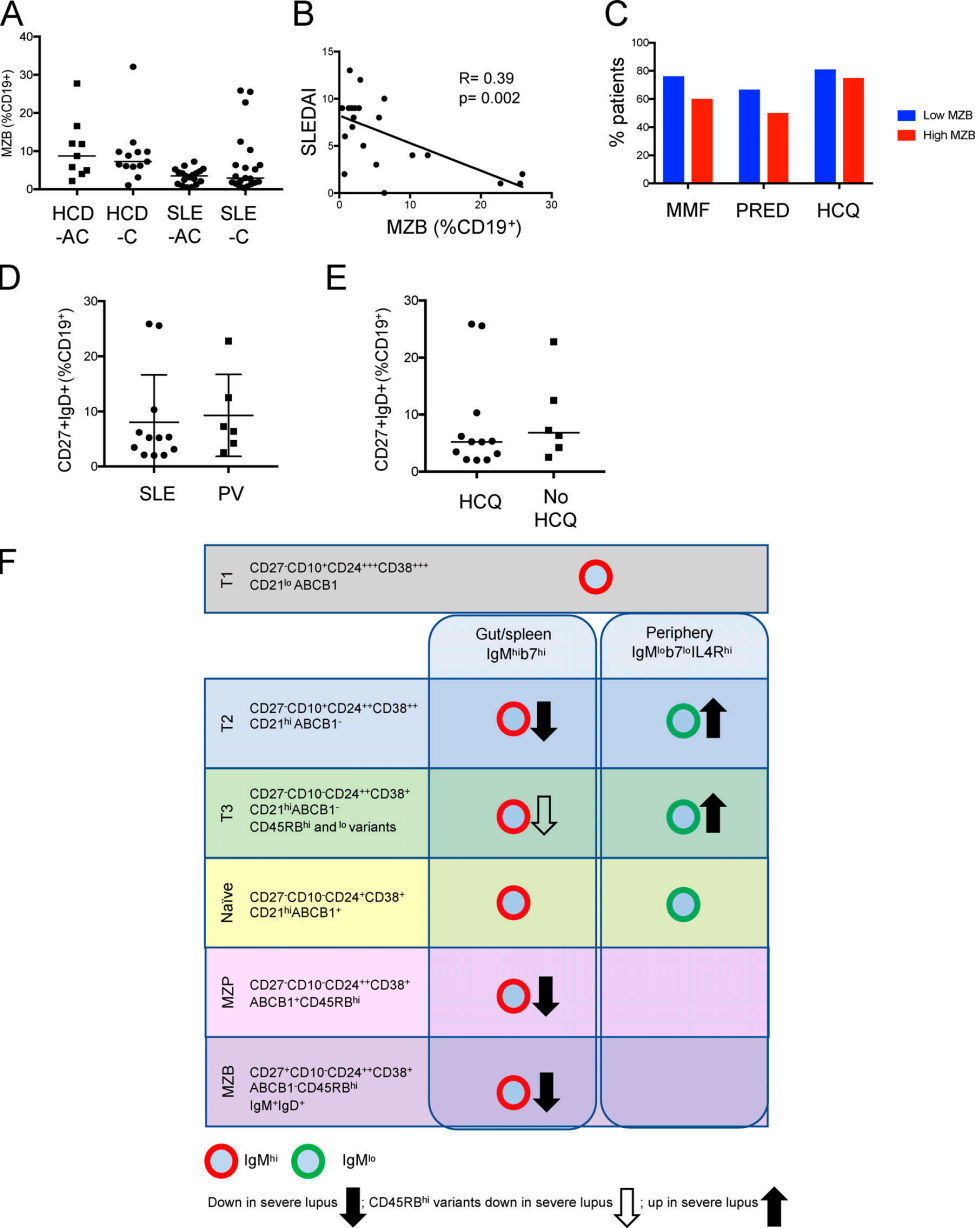
**The relationship between MZB counts and SLE clinical parameters and a proposed model of defective B cell development in SLE.** **(A)** Scatter plots of flow cytometry data demonstrating no difference in MZB frequency in African Caribbean (-AC) and Caucasian (-C) HCD and SLE patients (medians, Mann–Whitney test). **(B)** Correlation of MZB and SLE disease activity index (SLEDAI) score in Caucasian SLE patients (Spearman’s rank coefficient). **(C)** Bar graphs demonstrating the immunosuppressive burden of SLE patients with low MZB counts (<3.13% CD19^+^ cells) versus high MZB counts (>3.13% CD19^+^ cells), where 3.13% represents the median MZB value in all SLE patients. MMF, mycophenolate mofetil; HCQ, hydroxychloroquine; PRED, prednisolone. **(D)** Scatter plots of flow cytometry data demonstrate that PV patients taking mycophenolate mofetil and/or prednisolone did not have reduced MZB when compared with SLE patients on the same immunosuppressive medication (mean ± SD, unpaired *t* test). **(E)** Scatter plots of flow cytometry data demonstrate there was no difference in MZB counts in nonrenal SLE (OL) patients taking or not taking hydroxychloroquine (HCQ) therapy (medians, Mann–Whitney test). **(F)** Proposed model of MZB differentiation and alterations seen in severe SLE.

Flow cytometry was used to quantify B cell subsets in a cohort of 41 SLE patients and matched HCDs (Fig. S4, A and B). Reduced MZB frequency was seen in patients with SLE compared with HCDs (Fig. 6, A and B), and this was most marked in patients with LN compared with patients with other manifestations of SLE (other lupus subtypes [OLs]; Fig. 6 C). Reduced MZB frequency was not a feature of other autoimmune diseases studied (Figs. 6 C and S4 C), although it has been identified in patients with Sjögren’s disease (Roberts et al., 2014). We found that a relative reduction of MZB in patients with SLE was associated with a reduction of MZP (CD27^−^IgD^+^CD10^−^CD45RB^hi^ R123^−^; Fig. 6, D and E) and T3 CD45RB^hi^ cells (CD27^−^IgD^+^CD10^−^CD45RB^hi^R123^+^; Fig. 6, D and F). This was again most consistently observed in the LN patient cohort. The proportion of naive B cells (CD27^−^IgD^+^CD10^−^R123^lo^) was also diminished in SLE (Fig. 6 G), but CD45RB^lo^R123^hi^ cells were more frequent (Fig. 6 H). This population was further divided into T3 and aNAV cells by their expression of CD24 and CD38 (Fig. 6 I). CD45RB^lo^R123^hi^ cells were predominantly T3 cells (Fig. 6 J), although both subsets were increased in patients with LN (Fig. 6, K and L). Reduced frequency of MZB and precursor populations was not secondary to demographics or immunosuppressants but was more marked in more severe disease (Fig. S5, A–E). As previously reported, CD27^−^IgD^−^ DN cells were more abundant in LN (Fig. 6 M). These were predominantly CD24^lo^CD21^lo^ and therefore DN2, consistent with other studies (Jenks et al., 2018; Fig. 6, N and O).

MZB depletion in SLE is therefore associated with reduced frequency of MZP and T3 CD45RB^hi^ cells. This consolidates the concept of these cells as being in a developmental continuum in health and suggests that altered transitional B cell maturation or migration may result their depletion from the blood in SLE.

### IgM^hi^ β7 integrin^hi^ T2 cells are reduced in frequency in LN

**Figure 7.**
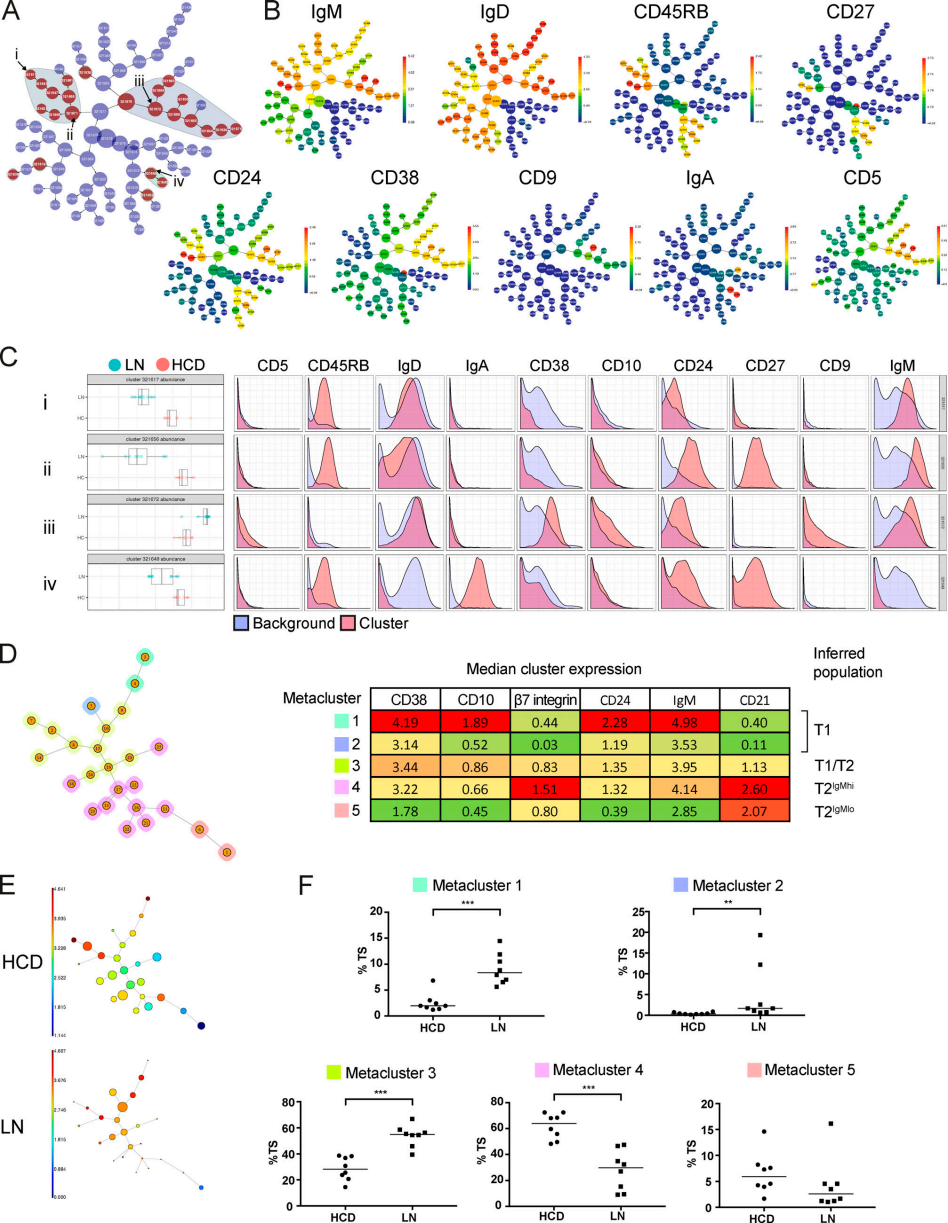
**IgM^hi^ β7 integrin^hi^ T2 cells are reduced in frequency in LN.** **(A)** CITRUS trees generated from CD19^+^ cells from HCDs (*n* = 8) and LN patients (*n* = 8) and clustered according to the expression of CD5, CD9, CD10, CD24, CD27, CD38, CD45RB, IgD, IgM, and IgA (see also Fig. S1, A–C; and Fig. S4 A). CITRUS trees represent nodes redundantly so that the most peripheral nodes contain cell populations that are progressively shared by more central nodes with the central node containing all events. Red nodes indicate significantly different population abundances between HCDs and LN patients. A gray background is automatically assigned to aggregates of significant nodes. Arrows and roman numerals indicate nodes further analyzed in C. **(B)** CITRUS trees demonstrating the median expression of the clustering panel markers in the nodes. **(C)** Histograms demonstrating the abundance and expression of panel markers in selected nodes. The identity of these nodes can be inferred as (i) MZP, (ii) MZB, (iii) TS B cells, and (iv) class-switched IgA memory. **(D)** Minimal spanning tree generated by FlowSOM from events exported from CITRUS node (iii) representing TS B cells identified in A and C. This generated five metaclusters; the identification of each could be inferred by median expression of panel markers in each metacluster. **(E)** Minimal spanning trees generated by FlowSOM plots demonstrating CD38 expression in a representative HCD and LN patient. Prominent skewing of TS B cell subpopulations is evident in LN. **(F)** Relative abundances of metaclusters as a percentage of TS B cells indicate reduced frequency of events within metacluster 4 corresponding to IgM^hi^ β7^hi^ TS B cells in LN and increased frequency of events in metaclusters 1, 2, and 3 corresponding to T1 cells (medians, Mann–Whitney test). **, P < 0.01; ***, P < 0.001.

To identify early stages of aberrant marginal zone lineage development in LN, we used mass cytometry to compare blood B cell subsets from LN patients and HCDs in an undirected way (Fig. S1, A–C). The automated clustering algorithm "cluster identification, characterization, and regression" (CITRUS) identified populations that differed significantly in abundance between LN and HCDs. The three main clusters of nodes (Fig. 7 A) can be identified by their relative expression of B cell lineage markers (Fig. 7, B and C). CD27^−^IgD^+^CD10^−^ CD45RB^hi^ (MZP) and CD27^+^IgD^+^CD10^−^ CD45RB^hi^ (MZB) cells were significantly reduced in LN patients (Fig. 7 C [i and ii]). TS B cells were more abundant (Fig. 7 C [iii]) while IgA class-switched cells were reduced in patients with LN (Fig. 7 C [iv]).

TS B cell subpopulations in HCDs and LN were quantified by FlowSOM (Fig. 7, D and E). Five metaclusters were identified representing subdivisions of T1 and T2 populations (Fig. 7 D). Inspection of minimal spanning trees demonstrated stark reduction of certain TS B cell subpopulations in LN (Fig. 7 E). Quantification of events within metaclusters from all donors revealed that T1 cells represented by metaclusters 1, 2, and 3 were more abundant in LN, while IgM^hi^ T2 cells with high expression of β7 integrin represented by metacluster 4 were markedly less abundant in LN (Fig. 7 F).

Reduced frequency of IgM^hi^ T2 cells with high expression of β7 integrin is therefore associated with MZB depletion in LN patients. This supports the association between MZB and IgM^hi^ T2 cells in health and implicates reduced access of these cells to GALT in the alteration of this developmental axis in patients with severe lupus (Fig. S5 F).

## Discussion

We have identified branches of human B cell lineage maturation that are evident from the T2 stage. An IgM^hi^ branch that expresses higher levels of β7 integrin and lower levels of IL4R compared with the IgM^lo^ branch is gut homing. Confirmation of differentiation through IgM^hi^ stages of differentiation from IgM^hi^ T2, including IgM^hi^CD45RB^hi^ T3 and naive B cell variants, to MZBs is gained from pseudotime analysis coupled with the observed concerted reduction of the stages in this sequence in patients with severe SLE (Fig. S5 F).

The reduced frequencies of gut-homing IgM^hi^ T2 cells in severe SLE further consolidates the role of GALT in early B cell fate decisions and supporting MZB development. Although it is not clear whether reduced frequency of cells in this pathway in blood reflects defects in differentiation, alternative homing patterns, or cell death, the absence of the gut-homing MZB maturational axis in blood in severe SLE affirms its existence in health. It is also not clear whether alteration of our proposed MZB differentiation pathway is a causative factor or a consequence of inflammation. The inverse correlation of MZB counts and disease activity may support the latter.

We have previously observed that human T2 cells are recruited into GALT, where they are activated by intestinal microbes (Vossenkämper et al., 2013). Here, we demonstrate that specifically the IgM^hi^ T2 subset of TS B cells is recruited into GALT, where they have a phenotype of activated cells, including expression of CD69 and CD80. The IgM^hi^ T2 subset is also enriched in retinoic acid and LPS-inducible genes, consistent with exposure to the microbiota. We show that the TLR9 agonist CpG that up-regulates IgM and Notch 2 in human TS B cells (Capolunghi et al., 2008; Guerrier et al., 2012) also up-regulates CD45RB on IgM^hi^ TS and IgM^hi^ naive cells. *PLD4*, a lupus risk allele and the most highly up-regulated gene in IgM^hi^ compared with IgM^lo^ TS B cells, is up-regulated along the developmental pathway to MZBs and limits responses to CpG (Gavin et al., 2018). This suggests that PLD4 defects could contribute to SLE pathogenesis by impacting an aspect of the development or function of IgM^hi^ TS B cells involving TLR9. Interestingly, *PLD4* is also expressed in the splenic marginal zone in mice (Yoshikawa et al., 2010), and *PLD4* knockout mice develop autoantibodies and immune complex–mediated renal damage similar to SLE with LN (Gavin et al., 2018). IgM^hi^ TS B cells also show a transcriptomic signature indicative of retinoic acid regulation that is a feature of GALT microenvironment. Together, these data suggest that innate signals and the gut environment impact the origin, fate, and function of IgM^hi^ TS B cells.

Consistent with proposed developmental continuum from the IgM^hi^ T2 stage through to MZBs, GALT is involved in MZB development, including a stage of receptor diversification in GALT GCs. However, supporting a relatively short-term transit coupled to differentiation, the frequencies of somatic mutations in MZBs are lower than those of memory B cells or plasma cells in the gut (Zhao et al., 2018). Together, these data suggest that GALT transit and GC occupancy are important but transient phases in IgM^hi^ T2 to MZB lineage progression. Pseudotime analysis also identified a population of B cells that appear to develop from MZBs and that are activated and more mature. It is possible that activation of MZBs might generate a novel population of effector or memory cells.

MZB differentiation is associated with distinctive gene expression changes and acquisition of the transcription factor ZEB2 (SIP1). ZEB2 has previously been identified as a component of a network including miR200 and TGF-β1 that can regulate cell fate decisions (Gregory et al., 2008; Guan et al., 2018). Activated TGFβ-1 is produced abundantly in the gut. It is possible that in addition to playing important roles in regulation of intestinal immunity as a switch factor for IgA and induction of regulatory T cells, it could also be involved in gut-associated MZB development by interactions with ZEB2 (Borsutzky et al., 2004; Chen et al., 2003).

Alteration of the MZB developmental pathway in the blood of severe SLE patients was accompanied by expanded T3, aNAV, and DN2 cell populations. Expansion of aNAV and DN2 populations is a product of excessive TLR7 and IFN-γ signaling. We were therefore interested in enrichment of IFN-γ–induced genes in IgM^lo^ TS B cells. Interestingly, the IFN-γ–regulated transcription factor KLF2 was transcriptionally up-regulated in IgM^lo^ TS B cells. KLF2 drives follicular B cell maturation in mice and its deletion results in an expansion of MZB cells. The role of KLF2 in human B cell development is not known; however, loss-of-function *KLF2* mutations along with *NOTCH2* mutations that increase the stability of the notch intracellular domain are the most commonly encountered mutations in human MZB cell lymphoma (Campos-Martín et al., 2017). This implicates KLF2 in human B cell fate decisions, suggests a role for IFN-γ in B cell development, and supports its proposed involvement of the imbalance of B cell subsets in LN. The role of IFN-γ in defective MZB maturation is also supported by reduction of this subset in patients with severe COVID-19, which is associated with elevated serum IFN-γ levels and extrafollicular B cell responses (Laing et al., 2020; Woodruff et al., 2020).

LN represents a severe lupus subtype associated with the worst clinical outcomes (Yap et al., 2012). B reg cell IL-10 responses associated with expression of CD80 and CD86 are defective in SLE (Blair et al., 2010), permitting aberrant T effector functions (Oleinika et al., 2019). In mice, B reg cells are IgM^hi^CD21^hi^CD23^hi^ T2 MZPs, and interaction with the gut microbiome is essential for their induction (Evans et al., 2007; Rosser et al., 2014). We have identified that IgM^hi^ TS B cells express CD80 in GALT and represent the predominant IL-10–producing TS B cell subset. Their reduction in LN may be synonymous with the loss of B reg IL-10 responses and associated with the lack of T cell regulation in SLE. MZBs confer immunity to encapsulated bacteria such as pneumococcus, and their reduction in LN may confer increased risk of such infections in SLE (Danza and Ruiz-Irastorza, 2013). This also reinforces the importance of pneumococcal vaccination in this patient cohort.

Undirected analysis of B cells in blood in LN compared with HCDs identified reduction in IgA memory B cells in LN. IgA deficiency can be observed in SLE, though the mechanism driving this is not known (Cassidy et al., 2007; Odineal and Gershwin, 2020). It is possible that lower expression of β7 integrin by B cells could contribute to IgA deficiency in SLE.

In summary, we identify an MZB maturation pathway that becomes evident at the T2 stage of B cell development and that is depleted in severe SLE. Traffic through GALT is a component of this pathway that is potentially linked to the induction of human IL-10–producing B reg cells (Rosser et al., 2014). Together, this affirms the importance of tissue microenvironments in shaping the B cell functional repertoire and maintaining health. Understanding the regulators of early B cell fate will be a key to resolving the disturbances in B cell function in severe SLE.

## Materials and methods

### Data and code availability

All raw and processed next-generation sequencing data have been deposited with GEO under accession numbers GSE163602 and GSE163493. Code is available on github (https://github.com/jspencer-lab/MZBFromT2).

### Experimental subject details

All blood and tissue samples were obtained from adults with research ethics committee (REC) approval and informed consent. SLE patients were recruited using the following criteria: (1) fulfilment of four or more revised American College of Rheumatology classification criteria, (2) ANA-positive, (3) biological (belimumab or rituximab) naive, and (4) immunosuppressive regimen does not include azathioprine or cyclophosphamide within 6 mo of sample collection due to the severe depletion of naive B cells by these medications. All LN patients had diagnostic confirmation by renal biopsy. Blood was obtained from SLE patients and HCDs (REC reference 11/LO/1433: Immune regulation in autoimmune rheumatic disease, London–City Road & Hampstead Research Ethics Committee). Paired gut biopsies and blood were obtained from individuals undergoing colonoscopies in whom no mucosal abnormality was detected (REC reference 11/LO/1274: Immunology the intestine; features associated with autoimmunity, London–Camberwell St Giles Research Ethics Committee). Samples of cells draining the gut and spleen via the hepatic portal vein were obtained from liver perfusion before transplantation (REC reference 09/H0802/100: The role of innate immune system in hepatic allograft outcome, Dulwich Ethics Committee). Patient demographic data can be found in Fig. S4, A–C.

### Methods

#### Sample processing

Blood samples were diluted 1:1 in RPMI-1640 containing 10% FCS, 100 U/ml penicillin, and 100 µg/ml streptomycin (RPMI-P/S). Diluted blood was then layered onto Ficoll and centrifuged for 25 min with brake and accelerator set to 0. The buffy coat layer was then removed, and cells were washed in RPMI-P/S. PBMCs isolated from patients undergoing colonoscopy was used fresh, while PBMCs used for the analysis of HCDs and patients with SLE, ulcerative colitis, granulomatosis with polyangiitis, and pemphigus vulgaris (PV) were cryopreserved in FCS + 10% DMSO. Mononuclear cells from gut were obtained by the removal of epithelial cells with 1 mM EDTA in HBSS containing 100 U/ml penicillin and 100 µg/ml streptomycin for 30 min. Collagenase digest was then used to generate a cell suspension using collagenase D (1 mg/ml) and DNase (10 U/ml) in RPMI-P/S for 1 h.

#### Mass cytometry

Three mass cytometry panels were used. The staining protocols were as follows. Panel 1: Cryopreserved cells were washed and rested in RPMI-P/S + 0.1 mg/ml DNase at 37 degrees for 45 min. B cells were then negatively enriched using a Miltenyi B cell isolation kit II. 4 × 10^6^ cells were then viability stained with 1 ml cisplatin 25 µM in 1× PBS. Cells were then washed in PBS containing 0.5% BSA with 2 mM EDTA (cell staining medium [CS-M]) and resuspended in 10 µl Fc receptor blocking solution and left for 10 min on ice. IgG staining was then performed in 100 µl staining volume for 30 min on ice. Cells were then washed in CS-M and resuspended in the pretitrated volume of antibody mastermix. The volume was then adjusted to 100 µl with CS-M, and cells were stained for 30 min on ice. Metal-tagged antibodies used are listed in Fig. S1 A. Cells were then washed twice in PBS and fixed overnight in 16% paraformaldehyde. The following day cells were washed in PBS and DNA was stained with 1 µM intercalatin in 500 µl permeabilization buffer at room temperature for 20 min. Cells were then washed twice in PBS and twice in Milli-Q water before being resuspended in Milli-Q water plus EQ beads to a concentration of 0.5 × 10^6^/ml and run on a Helios Mass cytometer.

Panel 2: As for panel 1, except cells were stained fresh, were not enriched, and 2 × 10^6^ cells were viability stained with 1 ml rhodium intercalator diluted in 1:500 in PBS for 20 min at room temperature. Metal-tagged antibodies used are listed in Fig. S1 A.

Panel 3: As for panel 1, except cells were not enriched and IgG staining was not performed. Metal-tagged antibodies used are listed in Fig. S1 A.

#### Analysis of mass cytometry data

FCS files were normalized using Nolan laboratory software (v0.3, available online at https://github.com/nolanlab/bead-normalization/releases). A representative pre and post normalization plot is shown in Fig. S1 B. Where files were concatenated, the Cytobank FCS File Concatenation Tool was used (available online at https://support.cytobank.org/hc/en-us/articles/206336147-FCS-file-concatenation-tool). Files were then loaded onto the Cytobank (https://mrc.cytobank.org/) and gated to identify live CD19^+^ B cells (Fig. S1 C).

For the analysis of HCD PBMCs in Fig. 1, viSNE was run on equal numbers of CD19^+^ events (*n* = 35,000) from each HCD (*n* = 10). SPADE was then run on the viSNE coordinates, and B cell subsets were identified by placing nodes into bubbles. The TS bubble was identified as CD27^−^IgD^+^CD24^+++/++^CD38^+++/++^. Events within the TS bubble were exported, a further viSNE was run using equal events (*n* = 3,535) and all panel markers except CD45, CD3, CD14, and class-switched isotypes IgA and IgG, which are not expressed by TS B cells. CD45 was excluded due to homogenous expression and lack of contribution to clustering. SPADE was then run on the viSNE coordinates, and TS B cell populations were defined as demonstrated in Fig. 1 B.

For the analysis of PBMCs and GALT-derived B cells in Fig. 2, equal numbers of CD19^+^ events (*n* = 118,934) from concatenated PBMC (*n* = 7) and GALT (*n* = 7) samples were used to run a viSNE using all markers except for CD45, CD3, and CD14. SPADE was then run on the viSNE coordinates and TS B cells identified as CD27^−^IgD^+^CD10^+^ nodes. Events within the TS bubble were then exported, and equal numbers of events (*n* = 4,520) were clustered using FlowSOM. CD10, CD24, CD38, and IgM as clustering channels to allow the undirected visualization of markers on TS B cell populations.

For the analysis of PBMCs from HCDs and SLE samples in Fig. 7, CITRUS was run using equal numbers of CD19^+^ events (*n* = 20,000) from HCDs (*n* = 8) and SLE patients (*n* = 8) and the following clustering channels: CD5, CD9, CD10, CD24, CD27, CD38, CD45RB, IgD, IgM, and IgA. Due to event sharing among CITRUS nodes, node 321,672 identified in Fig. 7, A and C (iii), contains all CD27^−^IgD^+^CD24^+++/++^CD38^+++/++^ events and was therefore used for analysis of TS B cells. All events from this node were exported, and FlowSOM was run using equal event sampling (*n* = 657) and using all marker channels except CD45, CD3, CD14, and IgA.

#### Flow cytometry and cell sorting

Cryopreserved cells used for flow cytometry were thawed and washed in RPMI-P/S and then rested at 37°C in RPMI-P/S + 0.1 mg/ml DNase for 45 min. Viability staining with Zombie aqua dye was performed using 100 µl 1:200 dilution in 1× PBS, or with DAPI 0.1 mg/ml diluted 1:1,000 and added before sample acquisition on the flow cytometer. Cells were stained on ice for 15 min with pretitrated concentrations of antibodies. Staining with R123 was performed for 10 min at a concentration of 6 µM, and cells were washed and chased for 3 h in RPMI-P/S. All samples were analyzed by a BD LSRFortessa (BD Biosciences). Anti-Mouse/Rat beads (BD Biosciences) were used for fluorescent compensation, and gates were set using appropriate isotype controls. Cell sorting was performed using a BD FACSAria (BD Biosciences), and live single CD19^+^ B cells were gated as follows: IgM^hi^ TS: CD27^−^IgD^+^CD10^+^IgM^hi^, IgM^lo^ TS: CD27^−^IgD^+^CD10^+^IgM^lo^; IgM^hi^ naive: CD27^−^IgD^+^CD10^−^IgM^hi^; IgM^lo^ naive: CD27^−^IgD^+^CD10^−^IgM^lo^; where IgM^hi^ and IgM^lo^ gates captured 30% of the highest and lowest IgM-expressing cells, respectively.

#### Cytokine detection

Fresh PBMCs were isolated from HCDs and incubated for 6 h at 37°C with 50 ng/ml PMA and 250 ng/ml ionomycin with GolgiPlug at a dilution of 1:1,000. Cells were then surface stained as above followed by fixation with Cytofast buffer (BioLegend). Cells were then washed twice and stained with resuspended conjugated antibodies in permeabilization/wash buffer (BioLegend) for 20 min at room temperature.

#### Cell culture and stimulation analysis

Sorted IgM^hi^ and IgM^lo^ TS and naive (CD27^−^IgD^+^CD10^−^) B cell subsets were plated onto a 96-well plate seeded with 2 × 10^4^ cells per well. Wells containing CD40L-expressing HEK cells were also seeded with 2 × 10^4^ irradiated HEK cells per well. Cells were then stimulated with CpG-ODN 2.5 µg/ml or anti-IgM 10 µg/ml. Proliferation assays were performed on cells stained with CellTrace violet as per the manufacturer’s guidelines. Cells were then stained and analyzed by flow cytometry as above.

#### Single-cell RNA-sequencing library preparation

Sorted cell populations were loaded onto a 10x Genomics Chromium Controller, and 5′ gene expression, VDJ, and ADT (for samples in Fig. 4) were prepared according to the manufacturer’s guidelines. Samples used in Fig. 3 were sequenced using an Illumina NextSeq 500 platform. Samples used in Fig. 4 were sequenced using an Illumina HiSeq 2500 High Output platform. The 10x Genomics Cell Ranger workflow was then used for transcript alignment and the generation of sparse matrices for downstream analysis.

#### CITE-seq antibody staining

Cryopreserved samples were thawed and sorted using the gating strategy in Fig. S3 A. Cells were then washed and stained in a CITE-seq antibody cocktail at a concentration of 8 µg/ml for 30 min on ice (Fig. S3 B). Cells were then washed three times before loading onto the 10x Chromium controller.

#### Single-cell sequencing analysis

The Seurat R package (vs 3.1.1) was used to filter data to remove cells with low numbers of RNA transcripts, doublets, and cells with high levels of mitochondrial transcripts indicative of cell death. Immunoglobulin variable genes were then removed from the dataset as well as cells with low expression of B cell genes *CD79A*, *CD79B*, *CD19*, or *MS4A1.* Data from IgM^hi^ and IgM^lo^ TS B cells were merged, and the data were transformed in accordance with the SCTransform workflow before UMAP-based reduction of dimensionality and PCA-based clustering to identify populations (Hafmeister and Satija, 2019 *Preprint*). Heatmaps were then created using select genes from the top 60 differentially expressed genes in each sample and dot plots and violin plots on selected genes. Data from sorted CD19^+^ cells from HCDs used for Fig. 4 and Fig. S3 were initially analyzed individually followed by an integrated analysis. Individual analysis was performed using the quality control steps as well as the removal of IGHV genes and non B cells as described above. Data were then normalized and scaled and UMAP run on a PCA generated using 2,000 variable genes. Overlay of ADT and gene signal, violin plots, and median expression of markers by UMAP clusters was used to identify which B cell subsets they corresponded to. For the integrated data analysis, data from three HCDs were filtered using the quality control steps as well as the removal of IGHV genes and non–B cells as described above. Data were then normalized using the SCTransform wrapper in Seurat followed by integration using The Satija Laboratory Integration and Label Transfer protocol (Butler et al., 2018), with 3,000 integration features. The 2,000 most variable genes were then used to perform PCA, and a 3D UMAP was obtained from this. Clusters were obtained using the FindNeighbors and FindClusters functions within Seurat using default parameters. The UMAP coordinates and cluster allocations were then used to run Slingshot (Street et al., 2018). Randomized downsampling of 50% was required to improve the performance of trajectory inference in Slingshot. ADT overlay of the UMAP plot was used to identify the cluster composed of CD27^−^IgD^+^CD38^hi^ cells that best represented TS B cells, and this was chosen as the starting point from which Slingshot would build trajectories. A heatmap was then created using genes of interest among the top 100 differentially expressed genes on the trajectory.

#### Quantitative RT-PCR

Quantitative RT-PCR was performed using Taqman Gene Expression Assays (FAM; Thermo Fisher Scientific) were used to quantify *CCR7* and *ITGB7* expression in cDNA from sorted IgM^hi^ and IgM^lo^ TS B cell subsets. Reactions were performed in duplicate and multiplexed with Eukaryotic 18S rRNA Endogenous Control (VIC). Samples were run on a QuantStudio 5 Real Time PCR System (Thermo Fisher Scientific). ΔCT was calculated using Thermo Fisher Connect software (available online at (https://apps.thermofisher.com/apps/spa/#/dataconnect).

### Quantification and statistical analysis

#### Flow cytometry and mass cytometry data

Flow cytometry data were visualized and gated using FlowJo v 10.6.1. Mass cytometry data were analyzed using Cytobank software.

#### Statistical analysis

GraphPad Prism version 7.0 was used for statistical analysis. Paired *t* tests or Wilcoxon tests were used to compare paired samples while unpaired *t* tests or Mann–Whitney tests were used for unpaired samples. Adjusted P values are represented as *, P ≤ 0.05; **, P ≤ 0.01; ***, P ≤ 0.001; ****, P ≤ 0.0001. All error bars show the mean ± SD.

### Online supplemental material

Fig. S1 illustrates antibody panels, normalization and gating strategy for mass cytometry, flow cytometry gating relating to Figs. 2 and 3, and a heatmap relating to Fig. 2 B. Fig. S2 illustrates the sorting strategy, 10x Genomics workflow, and validation of data in Fig. 3. Fig. S3 illustrates the sorting strategy and 10x Genomics workflow, identification of B cell subsets represented by UMAP clusters in 10x HCD 1 in Fig. 4, and 3D UMAP plots illustrating B cell subsets in 10x HCD 2 and 3. Fig. S4 provides demographic data for patients and healthy controls. Fig. S5 shows the relationship between MZB counts and SLE clinical parameters and also a model of B cell development that includes alterations observed in SLE. Video 1 shows rotation of the 3D UMAP plot demonstrating B cell subsets from 10x HCD1 as depicted in Fig. 4 A. Video 2 shows rotation of the 3D UMAP plot demonstrating B cell subsets from 10x HCD2 as demonstrated in Fig. S3 H. Video 3 shows rotation of the 3D UMAP plot demonstrating B cell subsets from 10x HCD3 as demonstrated in Fig. S3 I. Video 4 shows rotation of 3D UMAP plot as depicted in Fig. 4 G with overlay of IgM ADT signal demonstrating that the Slingshot trajectory passes through IgM^hi^ naive B cells.
